# Antimicrobial Resistance in Bacteria Isolated From Canine Urine Samples Submitted to a Veterinary Diagnostic Laboratory, Illinois, United States

**DOI:** 10.3389/fvets.2022.867784

**Published:** 2022-05-04

**Authors:** Setyo Yudhanto, Chien-Che Hung, Carol W. Maddox, Csaba Varga

**Affiliations:** ^1^Department of Pathobiology, College of Veterinary Medicine, University of Illinois Urbana Champaign, Urbana, IL, United States; ^2^Veterinary Diagnostic Laboratory, Department of Veterinary Clinical Medicine, College of Veterinary Medicine, University of Illinois Urbana Champaign, Urbana, IL, United States; ^3^Carl R. Woese Institute for Genomic Biology, University of Illinois Urbana-Champaign, Urbana, IL, United States

**Keywords:** urinary tract infection, bacteria, antimicrobial resistance, Illinois, dog, USA (America)

## Abstract

The emergence of antimicrobial resistance (AMR) in dogs constitutes a threat to animal and human health. There is a lack of studies in Illinois that evaluated the prevalence of AMR among urinary bacterial pathogens. In the study, we included 803 isolates (299 Gram-positive and 504 Gram-negative) that were isolated from 2,583 canine urine samples submitted to the Veterinary Diagnostic Laboratory, the University of Illinois between 2019 and 2020 from dogs suspected of urinary tract infections (UTI). The most common Gram-positive isolates included *Staphylococcus pseudintermedius* (17.93%), *Enterococcus faecalis* (9.46%), *Streptococcus canis* (6.10%), and *Enterococcus faecium* (3.74%), while Gram-negative isolates included *Escherichia coli* (45.58%), *Proteus mirabilis* (11.08%), *Klebsiella pneumoniae* (3.11%), and *Pseudomonas aeruginosa* (2.99%). Among the Gram-positive isolates, *Staphylococcus pseudintermedius* isolates showed a very high prevalence of resistance to penicillin (56.94%), a high prevalence of resistance to trimethoprim-sulfamethoxazole (31.94%), enrofloxacin (29.17%), and oxacillin (27.08%). Among Gram-negative bacteria, *Escherichia coli* isolates showed a high prevalence of resistance to ampicillin (31.42%). Considering the high prevalence of resistance to antimicrobials commonly used to treat UTI in dogs, urine samples should be collected for bacterial culture and susceptibility testing before treatment initiation to prevent treatment failures and the development of multidrug resistance. Given the possibility of zoonotic transmission of antimicrobial-resistant bacteria, veterinarians when treating UTI cases, should inform dog owners of the potential transmission risk.

## Introduction

The emergence of antimicrobial resistance (AMR) in bacteria isolated from dogs with urinary tract infections (UTI) is an important animal health and public health issue (1–3). Urinary tract infections with multidrug-resistant (MDR) bacteria increase morbidity, treatment failures, and therapeutic cost (4). Direct contact among humans and dogs favors the zoonotic transmission of MDR bacteria (5), posing a health risk to vulnerable populations, especially children, and immunocompromised persons (6). In the United States of America (US), previous studies showed that the most common sources of MDR bacteria of dogs included the respiratory tract, urinary tract, and skin (7–9). It was also estimated that UTI affects ~14% of dogs during their lifetime (10). The most common bacteria isolated from canine UTI were *Escherichia coli, Staphylococcus* sp., *Enterococcus* sp., *Proteus mirabilis, Pseudomonas aeruginosa, Streptococcus* sp., and *Klebsiella* sp. (11–13). Often, major urinary bacterial pathogens of dogs can be resistant to antimicrobials commonly used to treat UTI or to antimicrobials important for human medicine. Previous studies described resistance to carbapenems in *E. coli* isolates (14), and resistance to fluoroquinolones in *Pseudomonas aeruginosa* isolates (15, 16). Also, an increase in MDR methicillin-resistant *Staphylococcus pseudintermedius* (MRSP) isolated from dogs with UTI has been reported (17–19).

Best practices for UTI diagnosis and management in companion animals involve the bacterial culture and sensitivity testing of isolated pathogens from urine before starting treatment (10, 20). However, antimicrobial treatment of UTI is often started empirically to relieve clinical symptoms, without performing these practices (20, 21). While uncomplicated UTI in dogs resolve within 3–10 days of treatment (20), recurrent infections are difficult to treat with first-line antimicrobials (22). Without urine culture and antimicrobial susceptibility tests, treatment of recurrent UTI may lead to improper antimicrobial choices and the development of MDR bacteria (23). Additionally, treatment with broad-spectrum antimicrobials of UTI of dogs might increase selection for MDR bacteria (24).

Retrospective evaluation of the most common bacteria isolated from urine samples of dogs with suspected UTI and assessment of their AMR patterns can guide clinicians on their first-line drug choices to treat UTI (13). The International Society for Companion Animal Infectious Diseases (ISCAID) has published UTI treatment guidelines for dogs (20), which emphasize that the first-line empirical drug choice should be based on the local prevalence of bacterial pathogens and their resistance profiles. Overall, amoxicillin, amoxicillin-clavulanic acid, and trimethoprim-sulphonamides are considered as the first empirical antimicrobial choices for UTI treatment in dogs; meanwhile, nitrofurantoin, fluoroquinolones, and 3rd generation cephalosporins are only recommended if resistance to first-line antimicrobials is detected or the condition of the patient warrants it (20).

Although in dogs the UTI diagnosis and management guideline is available, it is known that the prevalence of urinary bacterial pathogens and their AMR patterns vary across regions (23). Several previous studies were conducted in the US describing the AMR patterns of bacteria of companion animals (8, 9, 15, 24–27); however, no data is available from Illinois. Therefore, our study aims to address this knowledge gap by describing the prevalence of major Gram-positive and Gram-negative bacteria isolated from canine urine samples submitted to the Veterinary Diagnostic Laboratory, University of Illinois, and evaluating their AMR patterns. The provided information will support Illinois and US veterinarians in their antimicrobial choices when treating UTIs to minimize treatment failures and reduce the emergence of MDR bacteria. This study also intends to raise awareness among companion animal veterinarians of the importance of performing bacterial culture and susceptibility tests before starting UTI treatment.

## Methods

### Data Source and Management

De-identified laboratory data were acquired for 2,583 canine urine samples submitted to the Veterinary Diagnostic Laboratory, University of Illinois, between 2019 and 2020 from suspected UTI cases for bacterial culture and AMR testing. Of the total samples, 1,439 were culture-negative or did not contain pathogens. The 1,144 culture-positive isolate data were reviewed for duplicates and missing values, and 803 isolates were kept for further analysis. The following variables were extracted from the laboratory records: de-identified sample ID, year of submission, bacterial species, and their antimicrobial susceptibility test results. Information on the collection method of urine samples was not available.

### Bacterial Culture and Identification

For each submission, 10–100 μl of urine or a swab containing urine was plated onto a Columbia agar with 5% sheep blood and a MacConkey agar plate. The culture plates were inoculated aerobically at 37°C, and the growth of bacteria was evaluated after 24 and 48 h of incubation. Representative colonies of individual bacterial species were selected and identified by matrix-assisted laser desorption ionization-time of flight mass spectrometry (MALDI-TOF MS) (Microflex®, Bruker, Germany) following the manufacturer's instruction. For bacterial identification, a MALDI-TOF score of ≥ 2.0 was considered confident to identify bacteria to the species level, and a score between 1.70 and 1.99 was considered as reliable to identify bacteria to the genus level. For scores lower than 1.70 the bacteria were not identified.

### Antimicrobial Susceptibility Testing and Analysis

Antimicrobial susceptibility testing was performed using the broth microdilution method following the Clinical and Laboratory Standards Institute's (CLSI) guidelines. The minimum inhibition concentration (MIC) value of individual antibiotics for each tested bacterial strain was tested by using commercially available Sensititre® COMPGPIF (for Gram-positive bacteria) or COMPGNIF (for Gram-negative bacteria) panel plates. In brief, bacterial isolates were first purified on Columbia agar with 5% sheep blood and incubated aerobically at 37°C for 24 h. A 1–100 μl bacterial suspension, depending on the bacterial species, was transferred into a tube containing 11 ml of Mueller-Hinton broth with or without lysed horse blood following the manufacturer's (Sensititre®, Remel Inc.) instructions. Fifty microliters of broth with bacteria were further seeded into the wells of Sensititre® GPIF or GNIF plates and incubated in a 35°C incubator. The MIC values were evaluated after 18–24 h of incubation. Antimicrobial susceptibility for each bacteria was determined based on the MIC breakpoints of the Vet01S CLSI guidelines (28). When the MIC breakpoints were not available in the veterinary specific CLSI guidelines, the human specific M100 CLSI was used (29). The breakpoints were labeled as no breakpoints (NB) when the MIC breakpoints were not available in these guidelines. Bacterial isolates were classified as susceptible, intermediate, or resistant. For descriptive and statistical analysis, the intermediate isolates were re-classified as resistant. We used the AMR data interpretation implemented in the European Union as a guideline (30), to classify the prevalence of AMR of isolates as: rare: <0.1%, very low: 0.1–1.0%, low: > 1.0–10.0%, moderate: > 10.0–20.0%, high: > 20.0–50.0%, very high: >50.0–70.0% and extremely high: > 70.0%. Besides, the MIC50 and MIC90 values were determined to identify the necessary minimum inhibitory concentrations that inhibit the growth of 50% and 90% of bacteria (**Tables 2**, **5**).

The AMR pattern was analyzed for both Gram-positive and Gram-negative bacterial groups. Hierarchical clustering dendrograms (heatmaps) were only constructed for bacteria with at least 30 isolates that had MIC breakpoints listed in the Vet01S or M100 CLSI guidelines. Isolates were categorized as MDR if they were resistant to at least one antimicrobial agent in at least three different antimicrobial classes and extensively drug-resistant (XDR) if they were resistant to all classes except 2 or less (31).

### Data Analysis

Statistical analyses were performed by using R Studio (Version 1.4.1106 2009-2021 RStudio, PBC) and STATA Intercooled (Version 14.2, Stata Corporation, College Station, TX) software. For each Gram-positive and Gram-negative bacteria, the prevalence of AMR to individual antimicrobials was computed by dividing the number of bacterial isolates resistant to an antimicrobial agent by the total number of bacterial isolates.

Hierarchical single-linkage clustering dendrograms (heatmaps) were constructed using the heatmap.2 package with ggplots and RColorBrewer libraries in R software to assess bacterial isolates in terms of their similarity in their AMR status. Ward's hierarchical clustering method with Euclidean distances was used (32).

In addition, a logistic regression analysis using a generalized linear model was conducted to identify differences in AMR to individual antimicrobials between two of the most prevalent bacteria in each of the Gram-positive and Gram-negative bacterial groups. In the first model, the outcome binomial variable represented whether the bacterial isolate was *Staphylococcus pseudintermedius* (yes=1) or *Streptococcus canis* (no=0), while the independent binomial variable was represented by the antimicrobial agents to which an isolate was resistant. The second model's outcome variable represented whether the isolate was *E. coli* (yes=1) or *Proteus mirabilis* (no=0). For all models, the odds ratio was the measure of effect, and a *p* ≤ 0.05 on the Wald χ2 test represented a statistically significant association.

## Results

### Description of Submissions

A total of 803 isolates were available for inclusion in this study, after eliminating duplicate isolates, and isolates with missing information. Of the total positive bacterial isolates on aerobic culture, 299 were Gram-positive, and 504 were Gram-negative. The most common Gram-positive isolates were *Staphylococcus pseudintermedius* (*n* = 144, 17.93%), *Enterococcus faecalis* (*n* = 76, 9.46%), *Streptococcus canis* (*n* = 49, 6.10%), and *Enterococcus faecium* (*n* = 30, 3.74%). The most common Gram-negative isolates were *Escherichia coli* (*n* = 366, 45.58%), *Proteus mirabilis* (*n* = 89, 11.08%), *Klebsiella pneumoniae* (*n* = 25, 3.11%), and *Pseudomonas aeruginosa* (*n* = 24, 2.99%).

### Antimicrobial Resistance of Gram-Positive Bacterial Isolates

*Staphylococcus pseudintermedius* isolates had a very high prevalence of resistance to penicillin (56.94%); a high resistance to doxycycline (48.61%), tetracycline (48.61%), minocycline (45.83%), ampicillin (36.11%), clindamycin (34.72%), erythromycin (34.03%), trimethoprim-sulfamethoxazole (31.94%), enrofloxacin (29.17%), cefovecin (27.78%), cefazolin (27.08%), cefpodoxime (27.08%), cephalothin (27.08%), amoxicillin-clavulanic acid (27.08%), oxacillin (27.08%), marbofloxacin (26.39%), pradofloxacin (26.39%), and gentamicin (20.14%); a moderate resistance to chloramphenicol (18.06%); a low resistance to nitrofurantoin (1.39%); and no resistance was observed to rifampin and vancomycin (Table 1). Among the *Staphylococcus pseudintermedius* isolates, 39 isolates (27.08%) had a MIC breakpoint ≥ 0.5 μg/ml to oxacillin that defines an isolate as MRSP (Methicillin-Resistant *Staphylococcus pseudintermedius*).

**Table 1:** The proportion of antimicrobial resistance in Gram-positive bacteria isolated from urine samples submitted to the Veterinary Diagnostic Laboratory, University of Illinois, College of Veterinary Medicine, 2019–2020.

**Antimicobial class**	**Antimicrobial agents**	***Staphylococcus pseudintermedius*** **(*****N*** **= 144)**		***Streptococcus canis*** **(*****N*** **= 49)**		***Enterococcus faecalis*** **(*****N*** **= 76)**		***Enterococcus faecium*** **(*****N*** **= 30)**	
		**MIC Breakpoint** ^**a**^ ** (S ≤ x μg/mL)**	***n*** ** (%)** ^**b**^	**MIC Breakpoint** ^**a**^ ** (S ≤ x μg/mL)**	***n*** ** (%)** ^**b**^	**MIC Breakpoint** ^**a**^ ** (S ≤ x μg/mL)**	***n*** ** (%)** ^**b**^	**MIC Breakpoint** ^**a**^ ** (S ≤ x μg/mL)**	***n*** ** (%)** ^**b**^
Ansamycins	RIF	1	0 (0)	NB	NB	1	67 (88.16)	1	21 (70)
Aminoglycosides/ Aminocyclitols	AMI	4	NI	4	NI	IR	IR	IR	IR
	GEN	4	29 (20.14)	NB	NB	IR	IR	IR	IR
β-Lactam combination agents	AUG2	0.25/0.12	39 (27.08)	NB	NB	NB	NB	NB	NB
Cephalosporins	FAZ	2	39 (27.08)	2	0 (0)	IR	IR	IR	IR
	FOV	0.5	40 (27.78)	0.12	0 (0)	IR	IR	IR	IR
	POD	2	39 (27.08)	2	0 (0)	IR	IR	IR	IR
	CEP	2	39 (27.08)	2	0 (0)	IR	IR	IR	IR
Carbapenems	IMI	NB	NB	NB	NB	NB	NB	NB	NB
Folate Pathway Antagonists	SXT	2	46 (31.94)	NB	NB	IR	IR	IR	IR
Fluoroquinolones	ENRO	0.5	42 (29.17)	0.5	32 (65.31)	NB	NB	NB	NB
	MAR	1	38 (26.39)	1	27 (55.1)	NB	NB	NB	NB
	PRA	0.25	38 (26.39)	NB	NB	NB	NB	NB	NB
Glycopeptides	VAN	4	0 (0)	1	0 (0)	4	0 (0)	4	0 (0)
Macrolides	ERY	0.5	49 (34.03)	0.25	5 (10.2)	0.5	50 (65.79)	0.5	30 (100)
Lincosamides	CLI	0.5	50 (34.72)	0.5	5 (10.2)	IR	IR	IR	IR
Nitrofurans	NIT	32	2 (1.39)	NB	NB	32	0 (0)	32	30 (100)
Penicillins	AMP	0.25	52 (36.11)	0.25	0 (0)	8	0 (0)	8	22 (73.33)
	PEN	0.12	82 (56.94)	0.25	0 (0)	8	0 (0)	8	23 (76.67
	OXA	0.25	39 (27.08)	NB	NB	NB	NB	NB	NB
Phenicols	CHL	8	26 (18.06)	4	NI	8	13 (17.11)	8	9 (30)
Tetracyclines	DOX	0.12	70 (48.61)	NB	NB	4	NI	4	NI
	TET	0.25	70 (48.61)	NB	NB	4	NI	4	NI
	MIN	0.5	66 (45.83)	NB	NB	4	NI	4	NI

*RIF, Rifampin; GEN, gentamicin; AUG2, amoxicillin-clavulanic acid; FAZ, cefazolin; FOV, cefovecin; POD, cefpodoxime; CEP, cephalothin; IMI, imipenem; SXT, trimethoprim-sulfamethoxazole; ERY, erythromycin; ENRO, enrofloxacin; MAR, marbofloxacin; PRA, pradofloxacin; VAN, vancomycin; CLI, clindamycin; NIT, nitrofurantoin; AMP, ampicillin; PEN, penicillin; OXA, oxacillin; CHL, chloramphenicol; DOX, doxycycline; TET, tetracycline; MIN, minocycline*.

a *Minimum inhibitory concentration (MIC) based on Vet01S and M100 Clinical Laboratory Standards Institute (CLSI) guidelines*.

b *Number and percentage of isolates resistant to antimicrobial; NB (No breakpoints), MIC breakpoint is not available in Vet01S and M100 CLSI guidelines; NI (Not interpretable), Test range do not contain the MIC breakpoint; IR, Intrinsic resistance*.

*Streptococcus canis* isolates had a very high prevalence of resistance to enrofloxacin (65.31%) and marbofloxacin (55.1%); and a moderate prevalence of resistance to erythromycin (10.2%) and clindamycin (10.2%). At the same time, no resistance was observed to cephalosporins, vancomycin, penicillin, and ampicillin (Table 1). In both *Enterococcus faecalis* and *Enterococcus faecium*, there were no breakpoints available for 14 out of 24 antimicrobials tested. Therefore, their susceptibility was only described for the ten remaining antimicrobials. Among the *Enterococcus faecalis* isolates, there was an extremely high prevalence of resistance to rifampin (88.16%) and a very high prevalence of resistance to erythromycin (65.79%). While in the *Enterococcus faecium* isolates, there was an extremely high prevalence of resistance to erythromycin (100%), nitrofurantoin (100%), penicillin (76.67%), and ampicillin (73.33%); and a very high prevalence of resistance to rifampin (70%) (Table 1).

In addition, susceptibility to amikacin in the case of *Staphylococcus pseudintermedius* and *Streptococcus canis* isolates; to chloramphenicol for *Streptococcus canis* isolates; and to the tetracyclines class for *Enterococcus faecalis* and *Enterococcus faecium* isolates were not interpreted because the MIC test range included on the plate wells did not contain the MIC value (e.g., MIC breakpoint) that defines a susceptible isolate (Table 2).

**Table 2:** The MIC50 and MIC90 values of the Gram-positive bacterial isolates.

**Antimicrobial agents**	***Staphylococcus pseudintermedius*** **(*****N*** **= 144)**			***Streptococcus canis*** **(*****N*** **= 49)**			***Enterococcus faecalis*** **(*****N*** **= 76)**			***Enterococcus faecium*** **(*****N*** **= 30)**			**Test range**
	**MIC 50**	**MIC 90**	**MIC range**	**MIC 50**	**MIC 90**	**MIC range**	**MIC 50**	**MIC 90**	**MIC range**	**MIC 50**	**MIC 90**	**MIC range**	
RIF	≤ 1	≤ 1	≤ 1, ≤ 1	≤ 1	≤ 1	≤ 1, 2	> 2	> 2	≤ 1, > 2	> 2	> 2	≤ 1, > 2	1–2
AMI	≤ 16	≤ 16	≤ 16, ≤ 16	> 32	> 32	≤ 16, > 32	IR	IR	IR	IR	IR	IR	16–32
GEN	≤ 4	16	≤ 1, 16	≤ 4	16	≤ 4, > 16	IR	IR	IR	IR	IR	IR	4–16
AUG2	≤ 0.25	4	≤ 0.25, > 8	≤ 0.25	≤ 0.25	≤ 0.25, ≤ 0.25	1	1	0.5, 2	> 8	> 8	≤ 0.25, > 8	0.25/0.12–8/4
FAZ	≤ 2	≤ 2	≤ 1, > 4	≤ 2	≤ 2	≤ 2, ≤ 2	IR	IR	IR	IR	IR	IR	2–4
FOV	0.25	> 8	≤ 0.06, > 8	≤ 0.06	≤ 0.06	≤ 0.06, 0.12	IR	IR	IR	IR	IR	IR	0.06–8
POD	≤ 2	> 8	≤ 2, > 8	≤ 2	≤ 2	≤ 2, ≤ 2	IR	IR	IR	IR	IR	IR	2–8
CEP	≤ 2	≤ 2	≤ 2, > 4	≤ 2	≤ 2	≤ 2, ≤ 2	IR	IR	IR	IR	IR	IR	2–4
IMI	≤ 1	≤ 1	≤ 1, > 4	≤ 1	≤ 1	≤ 1, ≤ 1	≤ 1	≤ 1	≤ 1, 2	> 4	> 4	≤ 1, > 4	1–4
SXT	≤ 2	> 4	≤ 0.5, > 4	≤ 2	≤ 2	≤ 2, ≤ 2	IR	IR	IR	IR	IR	IR	2/38–4/76
ENRO	≤ 0.25	> 4	≤ 0.25, > 4	1	2	≤ 0.25, 4	1	> 4	≤ 0.25, > 4	> 4	> 4	1, > 4	0.25–4
MAR	≤ 1	> 4	≤ 0.25, > 4	2	4	≤ 1, > 4	4	> 4	≤ 1, > 4	> 4	> 4	2, > 4	1–4
PRA	≤ 0.25	2	≤ 0.25, > 2	≤ 0.25	≤ 0.25	≤ 0.25, 1	0.5	> 2	≤ 0.25, > 2	> 2	> 2	≤ 0.25, > 2	0.25–2
VAN	≤ 1	≤ 1	≤ 1, ≤ 1	≤ 1	≤ 1	≤ 1, ≤ 1	≤ 1	2	≤ 1, 4	≤ 1	≤ 1	0.5, 2	1–16
ERY	≤ 0.25	> 4	≤ 0.25, > 4	≤ 0.25	> 4	≤ 0.25, > 4	1	> 4	≤ 0.25, > 4	> 4	> 4	1, > 4	0.25–4
CLI	≤ 0.5	> 4	≤ 0.5, > 4	≤ 0.5	≤ 4	≤ 0.5, > 4	IR	IR	IR	IR	IR	IR	0.5–4
NIT	≤ 16	≤ 16	≤ 16, 64	≤ 16	≤ 16	≤ 16, ≤ 32	≤ 16	≤ 16	≤ 16, 32	64	> 64	64, > 64	16–64
AMP	≤ 0.25	> 8	≤ 0.25, > 8	≤ 0.25	≤ 0.25	≤ 0.25, ≤ 0.25	1	1	0.5, 4	> 8	> 8	0.5, > 8	0.25–8
PEN	0.25	> 8	≤ 0.06, > 8	≤ 0.06	≤ 0.06	≤ 0.06, <0.06	4	4	2, 4	> 8	> 8	1, > 8	0.06–8
OXA	≤ 0.25	> 2	≤ 0.25, > 2	≤ 0.25	≤ 0.25	≤ 0.25, ≤ 0.25	> 2	> 2	> 2, > 2	> 2	> 2	> 2, > 2	0.25–2
CHL	≤ 8	> 32	≤ 8, > 32	≤ 8	≤ 8	≤ 8, ≤ 8	≤ 8	32	≤ 8, > 32	≤ 8	32	≤ 8, 32	8–32
DOX	≤ 0.12	> 0.5	≤ 0.12, > 0.5	0.25	> 0.5	≤ 0.12, > 0.5	0.25	> 5	≤ 0.12, > 0.5	> 0.5	> 0.5	≤ 0.12, > 0.5	0.12–0.5
TET	≤ 0.25	> 1	≤ 0.25, > 1	> 1	> 1	1, > 1	0.5	> 1	0.5, > 1	> 1	> 1	≤ 0.25, > 8	0.25–1
MIN	≤ 0.5	> 2	≤ 0.5, > 2	≤ 0.5	> 2	≤ 0.5, > 2	≤ 0.5	> 2	≤ 0.5, > 2	> 2	> 2	≤ 0.5, > 2	0.5–2

*RIF, Rifampin; GEN, gentamicin; AUG2, amoxicillin-clavulanic acid; FAZ, cefazolin; FOV, cefovecin; POD, cefpodoxime; CEP, cephalothin; IMI, imipenem; SXT, trimethoprim-sulfamethoxazole; ERY, erythromycin; ENRO, enrofloxacin; MAR, marbofloxacin; PRA, pradofloxacin; VAN, vancomycin; CLI, clindamycin; NIT, nitrofurantoin; AMP, ampicillin; PEN, penicillin; OXA, oxacillin; CHL, chloramphenicol; DOX, doxycycline; TET, tetracycline; MIN, minocycline; IR, Intrinsic resistance*.

The most common AMR patterns are presented in Table 3. Among *Staphylococcus pseudintermedius* isolates the most common AMR pattern was resistance to penicillin (15 isolates, 10.42%). Among the *Streptococcus canis* isolates, enrofloxacin-marbofloxacin (15 isolates, 30.61%) was the major AMR pattern. For the *Enterococcus faecalis* isolates, the main AMR pattern was erythromycin-rifampin-amikacin-gentamicin-amoxicillin clavulanic acid-cefazolin-cefovecin-cefpodoxime-cephalotin (36 isolates, 47.37%). While, among the *Enterococcus faecium* isolates ampicillin-erythromycin-nitrofurantoin-penicillin-rifampin-amikacin-gentamicin-amoxicillin clavulanic acid-cefazolin-cefovecin-cefpodoxime-cephalotin-trimethoprim sulfamethoxazole-clindamycin was the most common pattern (9 isolates, 30%).

**Table 3:** The most common antimicrobial resistance patterns in Gram-positive bacteria isolated from canine urine samples.

**Bacteria**	**Antimicrobial resistance patterns**^**a**^, ^**b**^	**Number of antimicrobial classes in pattern**	***n*** **(%)**
*Staphylococcus*	PEN	1	15 (10.42)
*pseudintermedius*	AUG2-AMP-FAZ-FOV-POD-CEP-CLI-DOX-ENRO-ERY-MAR-MIN-PEN-OXA-PRA-TET-SXT	8	10 (6.94)
	AUG2-AMP-FAZ-FOV-POD-CEP-CLI-DOX-ENRO-ERY-GEN-MAR-MIN-PEN-OXA-PRA-TET-SXT	9	7 (4.86)
	DOX-MIN-TET	1	7 (4.86)
	Susceptible	0	36 (25.00)
*Streptococcus canis*	ENRO-MAR	1	15 (30.61)
	ENRO	1	13 (26.53)
	MAR	1	7 (14.29)
	CLI-ENRO-ERY-MAR	3	4 (8.16)
	Susceptible	0	9 (18.37)
*Enterococcus faecalis*	ERY-RIF-AMI^*^-GEN^*^-AUG2^*^-FAZ^*^-FOV^*^-POD^*^-CEP^*^-SXT^*^-CLI^*^	7	36 (47.37)
	RIF-AMI^*^-GEN^*^-AUG2^*^-FAZ^*^-FOV^*^-POD^*^-CEP^*^-SXT^*^-CLI^*^	6	22 (28.95)
	CHL-ERY-RIF-AMI^*^-GEN^*^-AUG2^*^-FAZ^*^-FOV^*^-POD^*^-CEP^*^-SXT^*^-CLI^*^	8	7 (9.21)
*Enterococcus faecium*	AMP-ERY-NIT-PEN-RIF- AMI^*^-GEN^*^-AUG2^*^-FAZ^*^-FOV^*^-POD^*^-CEP^*^-SXT^*^-CLI^*^	9	9 (30)
	AMP-CHL-ERY-NIT-PEN-RIF- AMI^*^-GEN^*^-AUG2^*^-FAZ^*^-FOV^*^-POD^*^-CEP^*^-SXT^*^-CLI^*^	10	4 (13.33)

a *Resistance patterns to 24 antimicrobial agents from COMPGP1F Sensititre™ Gram-positive plate*.

b *RIF, Rifampin; GEN, gentamicin; AUG2, amoxicillin-clavulanic acid; FAZ, cefazolin; FOV, cefovecin; POD, cefpodoxime; CEP, cephalothin; IMI, imipenem; SXT, trimethoprim-sulfamethoxazole; ERY, erythromycin; ENRO, enrofloxacin; MAR, marbofloxacin; PRA, pradofloxacin; VAN, vancomycin; CLI, clindamycin; NIT, nitrofurantoin; AMP, ampicillin; PEN, penicillin; OXA, oxacillin; CHL, chloramphenicol; DOX, doxycycline; TET, tetracycline; MIN, minocycline*.

* *Intrinsic resistance*.

**Figure 1:**
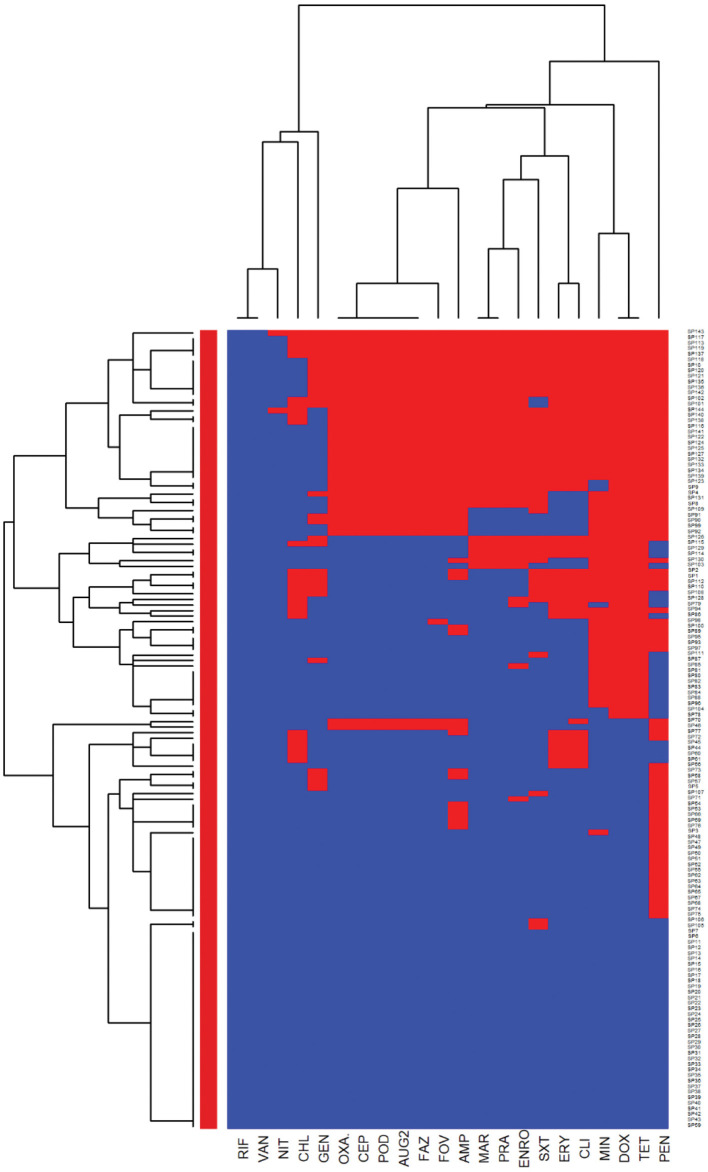
Hierarchical clustering dendrogram of antimicrobial resistance patterns in *Staphylococcus pseudintermedius* isolated from canine urine samples^a,b,c^. ^a^RIF, Rifampin; GEN, gentamicin; AUG2, amoxicillin-clavulanic acid; FAZ, cefazolin; FOV, cefovecin; POD, cefpodoxime; CEP, cephalothin; SXT, trimethoprim-sulfamethoxazole; ERY, erythromycin; ENRO, enrofloxacin; MAR, marbofloxacin; PRA, pradofloxacin; VAN, vancomycin; CLI, clindamycin; NIT, nitrofurantoin; AMP, ampicillin; PEN, penicillin; OXA, oxacillin; CHL, chloramphenicol; DOX, doxycycline; TET, tetracycline; MIN, minocycline. ^b^Heatmap generated by hierarchical clustering of the antimicrobial resistance determinants (columns) of bacterial isolates (rows). ^c^Red color, resistant; blue color, susceptible.

**Figure 2:**
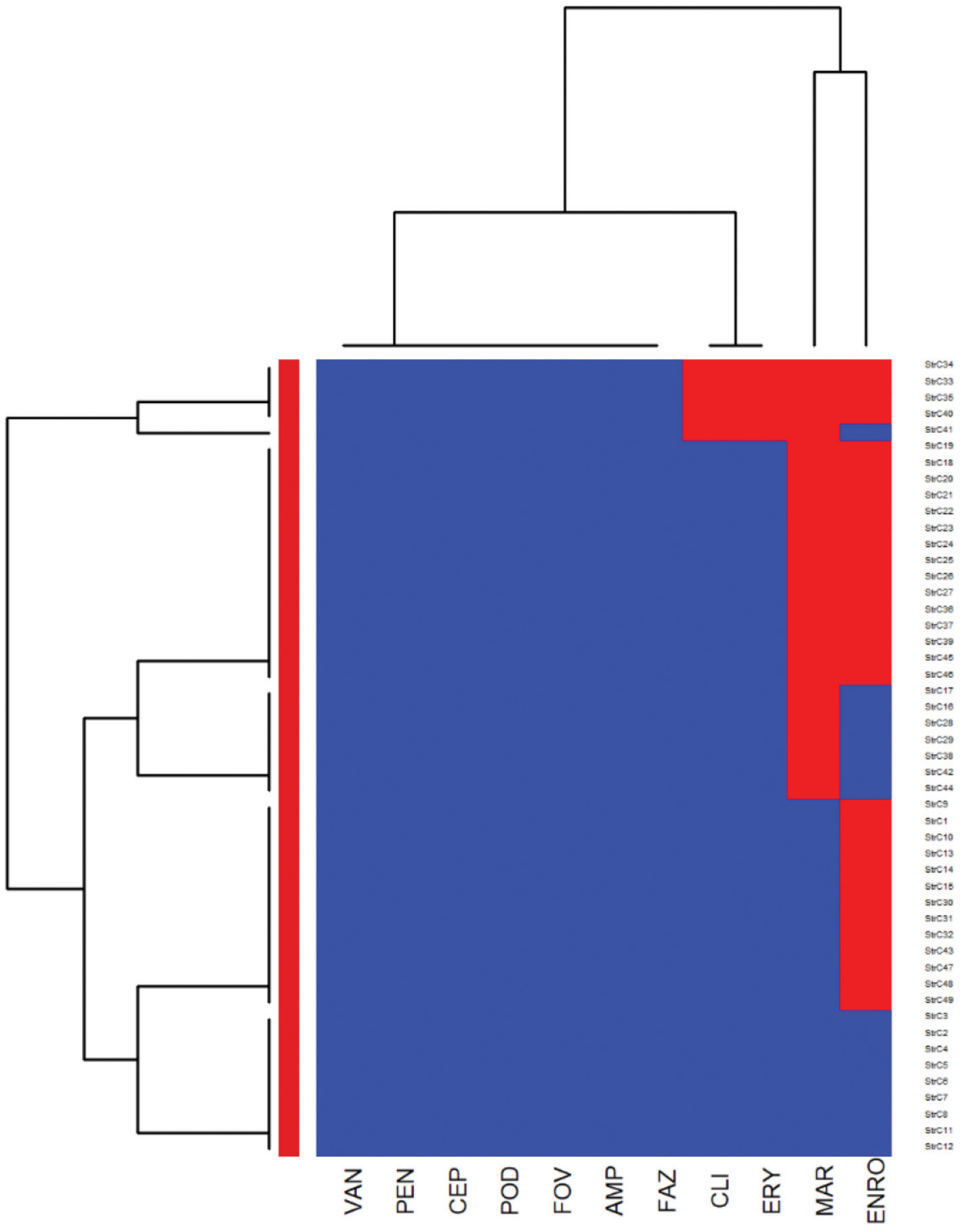
Hierarchical clustering dendrogram of antimicrobial resistance patterns in *Streptococcus canis* isolated from canine urine samples^a,b,c^. ^a^FAZ, Cefazolin; FOV, cefovecin; POD, cefpodoxime; CEP, cephalothin; ERY, erythromycin; ENRO, enrofloxacin; MAR, marbofloxacin; VAN, vancomycin; CLI, clindamycin; AMP, ampicillin; PEN, penicillin. ^b^Heatmap generated by hierarchical clustering of the antimicrobial resistance determinants (columns) of bacterial isolates (rows). ^c^Red color, resistant; blue color, susceptible.

The heatmaps (clustering dendrograms) for *Staphylococcus pseudintermedius* and *Streptococcus canis* isolates and their AMR patterns to individual antimicrobials are presented in Figures 1, 2. The clustering dendrograms were generated by using a hierarchical clustering method and were illustrated in heatmaps to evaluate the antimicrobial resistance determinants (columns) of bacterial isolates (rows). In the *Staphylococcus pseudintermedius* heatmap columns (Figure 1), several clusters of AMR patterns among antimicrobials tested were identified. A main cluster in the column included the cluster with a high to a very high prevalence of resistance to almost all antimicrobials tested. The second cluster in the heatmap column included susceptible isolates to rifampin and vancomycin, a low prevalence of resistance to nitrofurantoin, a moderate prevalence of resistance to gentamicin, and a high prevalence of resistance to chloramphenicol. While evaluating the clustering of bacterial isolates (rows), one main cluster of isolates was identified that was susceptible to all the tested antimicrobials and a second cluster included isolates that were resistant to most antimicrobials tested except for rifampin, vancomycin, and nitrofurantoin.

In the heatmap of the *Streptococcus canis* isolates (Figure 2), the first AMR cluster in the column included isolates with a very high prevalence of resistance to enrofloxacin and marbofloxacin. The second column cluster included susceptible isolates to ampicillin, cefazolin, cefovecin, cefpodoxime, cephalothin, penicillin, and vancomycin. In the bacterial isolates clustering (rows), a cluster of susceptible isolates to all interpretable antimicrobials was observed and a second cluster included isolates that were resistant to clindamycin, erythromycin, marbofloxacin, and enrofloxacin.

**Figure 3:**
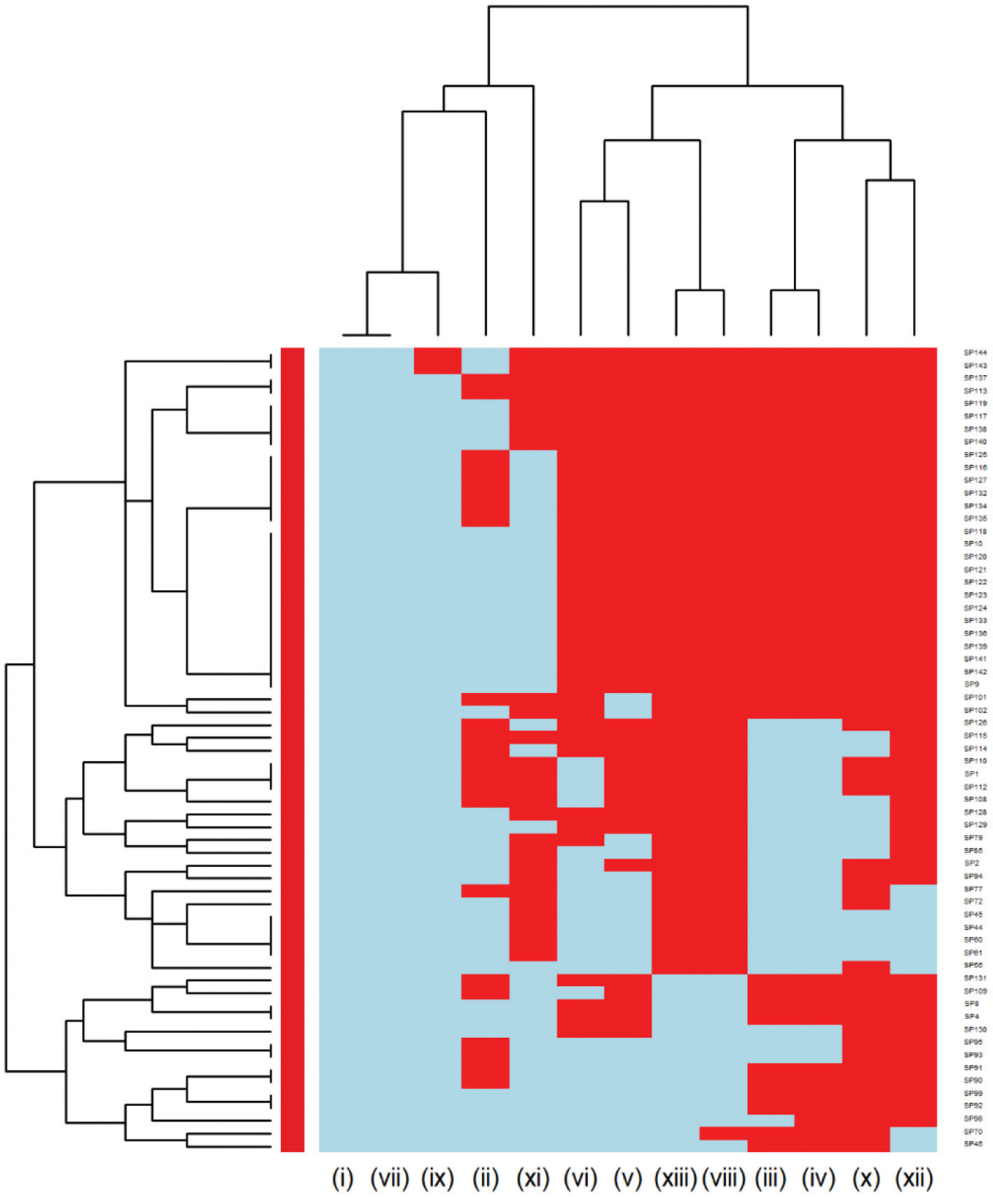
Hierarchical clustering dendrogram of multi drug resistance patterns in *Staphylococcus pseudintermedius* isolated from canine urine samples^a,b,c^. ^a^(i) Ansamycins; (ii) Aminoglycosides/Aminocyclitols; (iii) β-Lactam Combination Agents; (iv) Cephalosporins; (v) Folate Pathway Antagonists; (vi) Fluoroquinolones; (vii) Glycopeptides; (viii) Lincosamides; (ix) Nitrofurans; (x) Penicillins; (xi) Phenicols; (xii) Tetracyclines; (xiii) Macrolides. ^b^Heatmap generated by hierarchical clustering of the antimicrobial resistance determinants (columns) of bacterial isolates (rows). ^c^Red color, resistant; light blue color, susceptible.

Further analysis was conducted to investigate the MDR patterns (resistance to at least one antimicrobial agent in at least three different antimicrobial classes) of isolates (Figure 3). Of the 144 *Staphylococcus pseudintermedius* isolates, 63 were classified as MDR and none of the isolates was extensively drug-resistant (XDR). Inspecting the MDR heatmap's columns, it is seen that isolates were resistant to β-lactam combination agents, cephalosporins, fluoroquinolones, folate pathway antagonists, penicillins, and tetracyclines; and the second MDR pattern cluster included isolates resistant to phenicols, lincosamides, and macrolides. All isolates were susceptible to ansamycins, glycopeptides, and all isolates except two isolates were susceptible to nitrofurans. While inspecting the clustering of isolates (rows), the main cluster included a group of isolates that were resistant to β-lactam combination agents, cephalosporins, fluoroquinolones, folate pathway antagonists, penicillins, tetracyclines, lincosamides, and macrolides. For the *Streptococcus canis*, only 5 out of 49 isolates were classified as MDR, and no heatmap was constructed.

### Antimicrobial Resistance of Gram-Negative Bacterial Isolates

*Escherichia coli* isolates had a high prevalence of resistance to ampicillin (31.42%) and a low prevalence of resistance to piperacillin-tazobactam (3.28%), amikacin (2.73%), gentamicin (2.46%), and imipenem (0.27%). The other isolates showed a moderate prevalence of resistance (> 10.0–20.0%) to the remaining antimicrobials (Table 4). *Proteus mirabilis* isolates had a high prevalence of resistance to chloramphenicol (24.72%); a moderate prevalence of resistance to orbifloxacin (15.73%), trimethoprim-sulfamethoxazole (15.73%), and ampicillin (14.61%); a low prevalence of resistance to gentamicin (7.87%), enrofloxacin (7.87%), marbofloxacin (6.74), cefazolin (5.62%), imipenem (4.49%), amoxicillin-clavulanic acid (3.37%), cefovecin (2.25%), cefpodoxime (2.25%), and cephalexin (2.25%); and no resistance to amikacin, piperacillin-tazobactam, and ceftazidime.

**Table 4:** The proportion of antimicrobial resistance in Gram-negative bacteria isolated from urine samples submitted to the Veterinary Diagnostic Laboratory, University of Illinois College of Veterinary Medicine, 2019–2020.

**Antimicrobial class**	**Antimicrobial** ** agents**	***Escherichia coli*** **(*****N*** **= 366)**		***Proteus mirabilis*** **(*****N*** **= 89)**		***Klebsiella pneumoniae*** **(*****N*** **= 25)**		***Pseudomonas aeruginosa*** **(*****N*** **= 24)**	
		**MIC Breakpoint**^a^ **(S ≤ x μg/mL)**	***n*** **(%)**^b^	**MIC Breakpoint** ^a^ ** (S ≤ x μg/mL)**	***n*** **(%)**^b^	**MIC Breakpoint**^a^ **(S ≤ x μg/mL)**	***n*** **(%)**^b^	**MIC Breakpoint** ^a^ ** (S ≤ x μg/mL)**	***n*** ** (%)** ^b^
Aminoglycosides/ Aminocyclitols	AMI	4	10 (2.73)	16	0 (0)	16	0 (0)	4	1 (4.17)
	GEN	2	9 (2.46)	2	7 (7.87)	2	1 (4)	2	2 (8.33)
β-Lactam Combination Agents	AUG2	8	59 (16.12)	8	3 (3.37)	8	7 (28)	IR	IR
	PT4	8	12 (3.28)	8	0 (0)	8	8 (32)	8	2 (8.33)
Cephalosporins	FAZ	16	52 (14.21)	16	5 (5.62)	16	8 (32)	NB	NB
	FOV	2	56 (15.30)	2	2 (2.25)	NB	NB	NB	NB
	POD	2	53 (14.48)	2	2 (2.25)	2	8 (32)	NB	NB
	TAZ	4	41 (11.20)	4	0 (0)	4	8 (32)	8	0 (0)
	LEX	16	55 (15.03)	16	2 (2.25)	16	8 (32)	NB	NB
Carbapenems	IMI	1	1 (0.27)	1	4 (4.49)	1	0 (0)	2	1 (4.17)
Folate pathway antagonists	SXT	2	46 (12.57)	2	14 (15.73)	2	7 (28)	IR	IR
Fluoroquinolones	ENRO	0.5	41 (11.20)	0.5	7 (7.87)	0.5	9 (36)	NB	NB
	MAR	1	39 (10.66)	1	6 (6.74)	1	9 (36)	NB	NB
	ORB	1	51 (13.93)	1	14 (15.73)	1	9 (36)	NB	NB
	PRA	0.25	42 (11.48)	NB	NB	NB	NB	NB	NB
Penicillins	AMP	8	115 (31.42)	8	13 (14.61)	IR	IR	IR	IR
Phenicols	CHL	8	56 (15.30)	8	22 (24.72)	8	7 (28)	IR	IR
Tetracyclines	DOX	4	57 (15.57)	IR	IR	4	10 (40)	IR	IR
	TET	4	57 (15.57)	IR	IR	4	9 (36)	IR	IR

*AMI, Amikacin; GEN, gentamicin; AUG2, amoxicillin-clavulanic acid; PT4, piperacillin-tazobactam; FAZ, cefazolin; FOV, cefovecin; POD, cefpodoxime; TAZ, ceftazidime; LEX, cephalexin; IMI, imipenem; SXT, trimethoprim-sulfamethoxazole; ENRO, enrofloxacin; MAR, marbofloxacin; ORB, orbifloxacin; PRA, pradofloxacin; AMP, ampicillin; CHL, chloramphenicol; DOX, doxycycline; TET, tetracycline*.

a *Minimum inhibitory concentrations (MIC) based on Vet01S and M100 Clinical Laboratory Standards Institute (CLSI) guidelines*.

b *Number and percentage of isolates resistant to antimicrobial*.

*NB, No MIC breakpoint available on Vet01S and M100 CLSI guidelines; IR, Intrinsic resistance*.

*Klebsiella pneumoniae* isolates had a high prevalence of resistance to all antimicrobials tested, except for a low prevalence of resistance to gentamicin (4%) and no resistance to amikacin and imipenem. For the *Pseudomonas aeruginosa* isolates, the analysis was conducted for only 5 antimicrobial agents that had MIC breakpoints available (Table 5). Among *Pseudomonas aeruginosa* isolates, there was a low prevalence of resistance to gentamicin (8.33%), piperacillin-tazobactam (8.33%), amikacin (4.17%), and imipenem (4.17%), and all isolates were susceptible to ceftazidime.

**Table 5:** The MIC50, MIC90, and MIC range values of the Gram-negative bacterial isolates.

**Antimicrobial agents**	***Escherichia coli*** **(*****N =*** **366)**			***Proteus mirabilis*** **(*****N =*** **89)**			***Klebsiella pneumoniae*** **(*****N =*** **25)**			***Pseudomonas aeruginosa*** **(*****N =*** **24)**			**Test** ** range**
	**MIC 50**	**MIC 90**	**MIC range**	**MIC 50**	**MIC 90**	**MIC** ** range**	**MIC 50**	**MIC 90**	**MIC range**	**MIC 50**	**MIC 90**	**MIC range**	
AMI	≤ 4	≤ 4	≤ 4, 16	≤ 4	≤ 4	≤ 4, 8	≤ 4	≤ 4	≤ 4, ≤ 4	≤ 4	≤ 4	≤ 4, ≤ 4	4–32
GEN	0.5	1	≤ 0.25, > 8	1	2	≤ 0.25, > 8	≤ 0.25	0.5	≤ 0.25, > 8	1	2	≤ 0.25, 4	0.25–8
AUG2	4	> 8	1, > 8	1	2	≤ 0.25, > 8	2	> 8	2, > 8	IR	IR	IR	0.25/0.12–8/4
PT4	≤ 8	≤ 8	≤ 8, > 64	≤ 8	≤ 8	≤ 8, ≤ 8	≤ 8	> 64	≤ 8, > 64	≤ 8	≤ 8	≤ 8, 16	8/4–64/4
FAZ	2	> 32	≤ 1, > 32	≤ 1	8	≤ 1, > 32	2	> 32	≤ 1, > 32	> 32	> 32	> 32, > 32	1–32
FOV	1	> 8	≤ 0.25, >8	≤ 0.25	0.5	≤ 0.25, > 8	0.5	> 8	0.5, > 8	> 8	> 8	> 8, > 8	0.25–8
POD	≤ 1	> 8	≤ 1, > 8	≤ 1	≤ 1	≤ 1, > 8	≤ 1	> 8	≤ 1, > 8	> 8	> 8	> 8, >8	1–8
TAZ	≤ 4	8	≤ 4, > 16	≤ 4	≤ 4	≤ 4, ≤ 4	≤ 4	> 16	≤ 4, > 16	≤ 4	≤ 4	≤ 4, 8	4–16
LEX	8	> 16	2, > 16	8	16	≤ 0.5, > 16	4	> 16	4, > 256	> 16	> 16	> 16, > 16	0.5–16
IMI	≤ 1	≤ 1	≤ 1, 2	≤ 1	≤ 1	≤ 1, 8	≤ 1	≤ 1	≤ 1, ≤ 1	≤ 1	2	≤ 1, 8	1–8
SXT	≤ 0.5	> 4	≤ 0.5, > 4	≤ 0.5	> 4	≤ 0.5, > 4	≤ 0.5	> 4	≤ 0.5, > 128	IR	IR	IR	0.5/9.5–4/76
ENRO	≤ 0.12	> 4	≤ 0.12, > 4	≤ 0.12	0.5	≤ 0.12, > 4	≤ 0.12	> 4	≤ 0.12, > 4	0.5	> 4	≤ 0.12, > 4	0.12–4
MAR	≤ 0.12	> 4	≤ 0.12, > 4	≤ 0.12	0.25	≤ 0.12, > 4	≤ 0.12	> 4	≤ 0.12, > 4	0.25	4	≤ 0.12, > 4	0.12–4
ORB	≤ 1	> 8	≤ 1, > 8	≤ 1	4	≤ 1, > 8	≤ 1	> 8	≤ 1, > 8	2	> 8	≤ 1, > 8	1–8
PRA	≤ 0.25	2	≤ 0.25, > 2	≤ 0.25	0.5	≤ 0.25, > 2	≤ 0.25	> 2	≤ 0.25, > 2	≤ 0.25	> 2	≤ 0.25, > 2	0.25–2
AMP	4	> 8	1, > 8	1	> 8	≤ 0.25, > 8	IR	IR	IR	IR	IR	IR	0.25–8
CHL	8	16	≤ 2, > 32	8	32	≤ 2, > 32	4	> 32	≤ 2, > 32	IR	IR	IR	2–32
DOX	2	> 8	≤ 0.25, > 8	IR	IR	IR	2	> 8	1, > 8	IR	IR	IR	0.25–8
TET	≤ 4	> 16	≤ 4, > 16	IR	IR	IR	≤ 4	> 16	≤ 4, > 128	IR	IR	IR	4–16

*AMI, Amikacin; GEN, gentamicin; AUG2, amoxicillin-clavulanic acid; PT4, piperacillin-tazobactam; FAZ, cefazolin; FOV, cefovecin; POD, cefpodoxime; TAZ, ceftazidime; LEX, cephalexin; IMI, imipenem; SXT, trimethoprim-sulfamethoxazole; ENRO, enrofloxacin; MAR, marbofloxacin; ORB, orbifloxacin; PRA, pradofloxacin; AMP, ampicillin; CHL, chloramphenicol; DOX, doxycycline; TET, tetracycline; IR, Intrinsic resistance*.

The most frequent AMR pattern in *Escherichia coli* isolates were isolates resistance to ampicillin (18 isolates, 4.92%), chloramphenicol (16 isolates, 4.37%), and amoxicillin-clavulanic acid-ampicillin-cefazolin-cefovecin-cefpodoxime-ceftazidime-cephalexin (10 isolates, 2.73%). Among the *Proteus mirabilis* isolates, the most common AMR patterns observed was resistance to doxycycline-tetracycline (54 isolates, 60.67%) and chloramphenicol-doxycycline-tetracycline (12 isolates, 13.48 %) (Table 6).

**Table 6:** The most common antimicrobial resistance patterns of Gram-negative bacteria isolated from canine urine samples.

**Bacteria**	**Antimicrobial resistance patterns**^**a**^, ^**b**^	**Number of antimicrobial classes in pattern**	***n*** ** (%)**
*Escherichia coli*	AMP	1	18 (4.92)
	CHL	1	16 (4.37)
	AUG2-AMP-FAZ-FOV-POD-TAZ-LEX	3	10 (2.73)
	DOX-TET	1	7 (1.91)
	AMP-CHL-DOX-ENRO-MAR-ORB-PRA-TET-SXT	5	5 (1.37)
	Susceptible	0	205 (56.01)
*Proteus mirabilis*	DOX^*^-TET^*^	1	54 (60.67)
	CHL-DOX^*^-TET^*^	2	12 (13.48)
	ORB-DOX^*^-TET^*^	2	4 (4.49)
*Klebsiella pneumoniae*	AMP^*^	1	14 (56)
	AUG2-AMP^*^-FAZ-FOV-POD-TAZ-LEX-CHL-DOX-ENRO-MAR-ORB-PT4-PRA-TET-SXT	8	5 (20)
*Pseudomonas aeruginosa*	AUG2^*^-SXT^*^-AMP^*^-CHL^*^-DOX^*^-TET^*^	5	20 (83.33)

a *Resistance to 19 antimicrobial agents from COMPGN1F Sensititre™ Gram-negative plate*.

b *AMI, Amikacin; GEN, gentamicin; AUG2, amoxicillin-clavulanic acid; PT4, piperacillin-tazobactam; FAZ, cefazolin; FOV, cefovecin; POD, cefpodoxime; TAZ, ceftazidime; LEX, cephalexin; IMI, imipenem; SXT, trimethoprim-sulfamethoxazole; ENRO, enrofloxacin; MAR, marbofloxacin; ORB, orbifloxacin; PRA, pradofloxacin; AMP, ampicillin; CHL, chloramphenicol; DOX, doxycycline; TET, tetracycline*.

* *Intrinsic resistance*.

**Figure 4:**
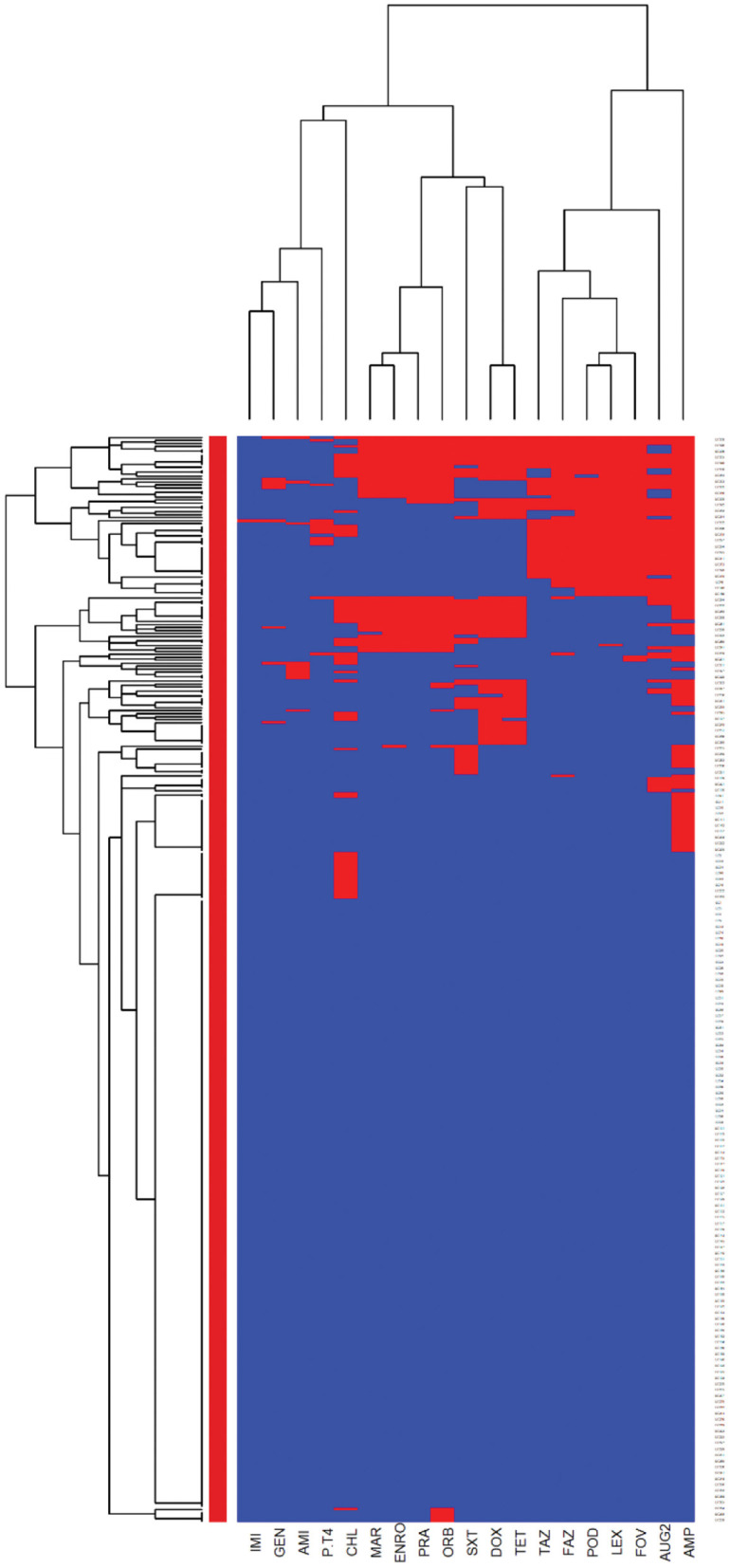
Antimicrobial resistance clustering dendrogram of *Escherichia coli* isolated from canine urine samples^a,b,c^. ^a^AMI, Amikacin; GEN, gentamicin; AUG2, amoxicillin-clavulanic acid; PT4, piperacillin-tazobactam; FAZ, cefazolin; FOV, cefovecin; POD, cefpodoxime; TAZ, ceftazidime; LEX, cephalexin; IMI, imipenem; SXT, trimethoprim-sulfamethoxazole; ENRO, enrofloxacin; MAR, marbofloxacin; ORB, orbifloxacin; PRA, pradofloxacin; AMP, ampicillin; CHL, chloramphenicol; DOX, doxycycline; TET, tetracycline. ^b^Red color, resistant; blue color, susceptible. ^c^Heatmap generated by hierarchical clustering of the antimicrobial resistance determinants (columns) of bacterial isolates (rows).

**Figure 5:**
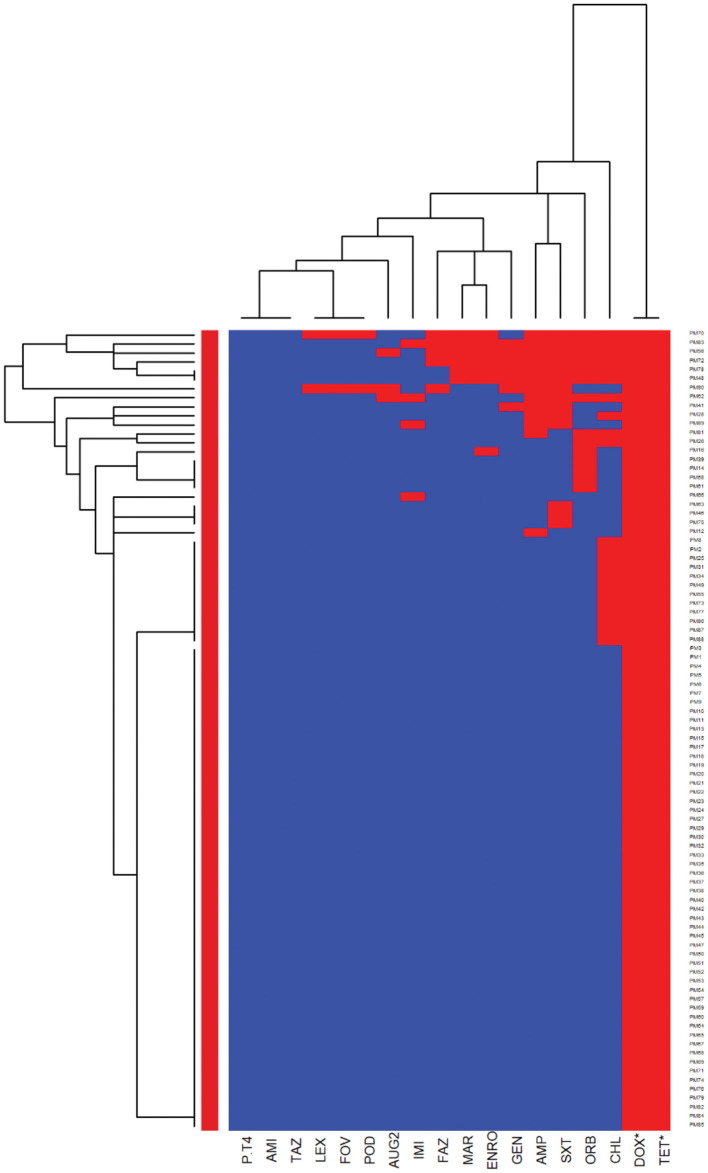
Antimicrobial resistance clustering dendrogram of *Proteus mirabilis* isolated from canine urine samples^a,b,c^. ^a^AMI, Amikacin; GEN, gentamicin; AUG2, amoxicillin-clavulanic acid; PT4, piperacillin-tazobactam; FAZ, cefazolin; FOV, cefovecin; POD, cefpodoxime; TAZ, ceftazidime; LEX, cephalexin; IMI, imipenem; SXT, trimethoprim-sulfamethoxazole; ENRO, enrofloxacin; MAR, marbofloxacin; ORB, orbifloxacin; AMP, ampicillin; CHL, chloramphenicol; DOX, doxycycline; TET, tetracycline. *Intrinsic resistance. ^b^Heatmap generated by hierarchical clustering of the antimicrobial resistance determinants (columns) of bacterial isolates (rows). ^c^Red color, resistant; blue color, susceptible.

The *E. coli* and *Proteus mirabilis* AMR heatmaps are presented in Figures 4, 5. In the *E. coli* heatmap (Figure 4), the main AMR cluster (columns) included a moderate prevalence of resistance to cephalexin, cefpodoxime, cefovecin, cefazolin, ceftazidime, amoxicillin-clavulanic acid, and a high prevalence of resistance to ampicillin. The second cluster included a moderate prevalence of resistance to tetracycline, doxycycline, trimethoprim-sulfamethoxazole, marbofloxacin, enrofloxacin, pradofloxacin, and orbifloxacin. Additionally, the third cluster included isolates that had a low prevalence of resistance to piperacillin-tazobactam, amikacin, gentamicin, and imipenem. Meanwhile, when evaluating clustering among *E. coli* isolates (rows), the main cluster was the cluster in which the isolates were susceptible to all antimicrobials tested; and a second cluster included isolates that were resistant to several antimicrobials. In the *Proteus mirabilis* heatmap (Figure 5), the main AMR clustering pattern (columns) included patterns of a high prevalence of resistance to chloramphenicol; a moderate prevalence of resistance to trimethoprim-sulfamethoxazole, orbifloxacin, and ampicillin; a low prevalence of resistance to gentamicin, amoxicillin-clavulanic acid, cefazolin, cefovecin, cefpodoxime, cephalexin, imipenem, enrofloxacin, and marbofloxacin; and susceptible to amikacin, piperacillin-tazobactam, and ceftazidime. A second AMR pattern cluster included intrinsic resistance to doxycycline and tetracycline. While inspecting the *Proteus mirabilis* clustering of isolates (rows), the main cluster included isolates that were susceptible to all antimicrobials except resistance to doxycycline and tetracycline (intrinsic resistance) and a cluster of isolates that were resistant to several antimicrobials.

**Figure 6:**
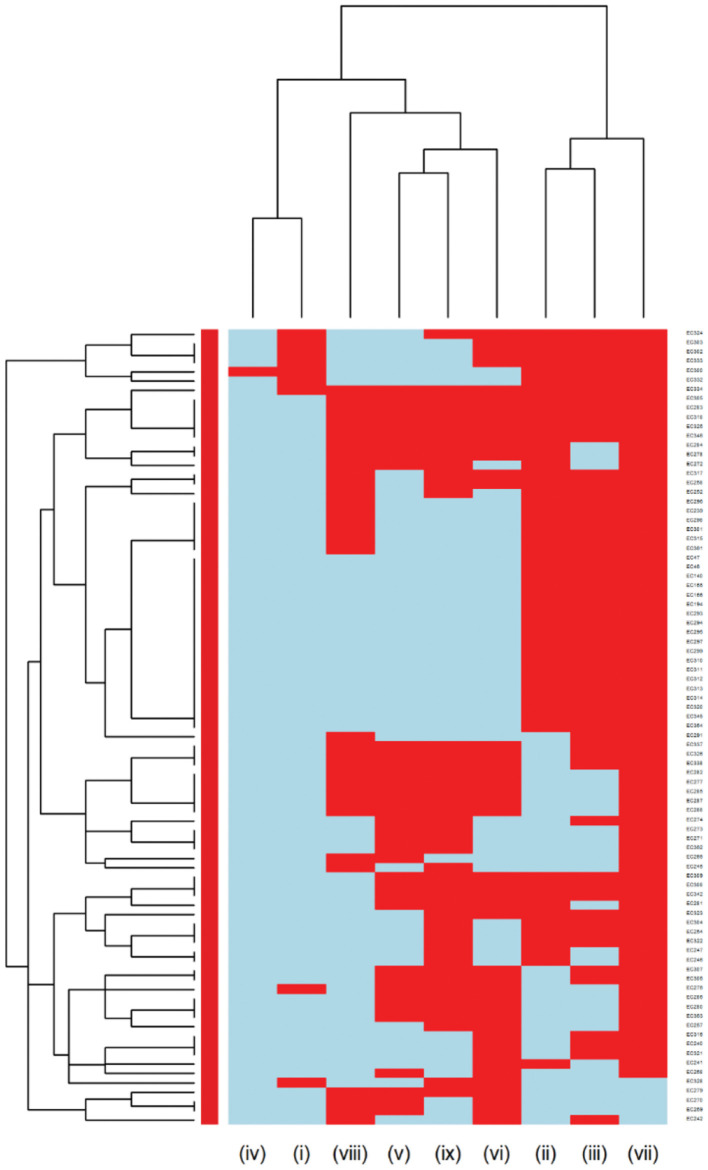
Hierarchical clustering dendrogram of multidrug resistance patterns in *E. coli* isolated from canine urine samples^a,b,c^. ^a^(i) Aminoglycosides/Aminocyclitols; (ii) β-Lactam Combination Agents; (iii) Cephalosporins; (iv) Carbapenems; (v) Folate Pathway Antagonists; (vi) Fluoroquinolones; (vii) Penicillins; (viii) Phenicols; (ix) Tetracyclines. ^b^Heatmap generated by hierarchical clustering of the antimicrobial resistance determinants (columns) of bacterial isolates (rows). ^c^Red color, resistant; light blue color, susceptible.

Multidrug resistance (resistance to at least one agent in at least 3 antimicrobial classes) was detected in 85 out of 366 *E. coli* isolates and 6 of the isolates were XDR (resistance to at least one agent in all but two or fewer antimicrobial classes). The MDR heatmap identified two AMR clusters (columns), the first one included resistance to penicillins, β-lactam combination agents, and cephalosporins classes, and the second cluster included resistance to phenicols, fluoroquinolones, folate pathway antagonists, and tetracyclines classes. One other AMR cluster included susceptibility to carbapenems and aminoglycosides classes (Figure 6). In the *Proteus mirabilis* isolates, 16 of the isolates were resistant to three or more antimicrobial classes.

### Assessing Differences in AMR Between the Two Most Common Gram-Positive Bacteria

Logistic regression models were constructed to compare AMR patterns among the two most frequent bacteria in each Gram-positive and Gram-negative group. In Gram-positive bacteria, the *Staphylococcus pseudintermedius* isolates were compared to *Streptococcus canis* isolates, due to the limited MIC breakpoints available for *Enterococcus* sp. isolates. When compared to *Streptococcus canis* isolates, *Staphylococcus pseudintermedius* had lower odds of resistance to enrofloxacin (OR = 0.22, *p* <0.001) and marbofloxacin (OR = 0.29, *p* < 0.001). The odds of resistance to erythromycin (OR = 4.54, *p* = 0.002) and clindamycin (OR = 4.68, *p* = 0.002) among *Staphylococcus pseudintermedius* isolates were higher than *Streptococcus canis* isolates (Supplementary Table 1).

### Assessing Differences in AMR Between the Two Most Common Gram-Negative Bacteria

Among Gram-negative bacteria, *E. coli* isolates were compared to *Proteus mirabilis* isolates (Supplementary Table 2). The odds of being resistant to cefovecin (OR = 7.86, *p* = 0.005), cephalexin (OR = 7.69, *p* = 0.005), cefpodoxime (OR = 7.37, *p* = 0.006), amoxicillin-clavulanic acid (OR = 5.51, *p* = 0.005), and ampicillin (OR = 2.68, *p* = 0.002) were significantly higher among *E. coli* isolates compared to *Proteus mirabilis* isolates. In contrast, the odds of resistance to chloramphenicol (OR = 0.55, *p* = 0.04), gentamicin (OR = 0.3, *p* = 0.02), and imipenem (OR = 0.06, *p* = 0.01) were lower in *E. coli* isolates compared to *Proteus mirabilis* isolates (Supplementary Table 2). In this comparison, tetracyclines were not included due to the known intrinsic resistance of *Proteus mirabilis* to these antimicrobials.

## Discussion

This study examined the prevalence and the AMR patterns of major bacteria isolated from canine urine samples over 2 years, submitted from suspected UTI cases to the Veterinary Diagnostic Laboratory, University of Illinois, a referral laboratory receiving samples from Illinois and other states in the USA. Antimicrobial susceptibility profiles of the main Gram-negative and Gram-positive bacterial isolates were determined using the broth microdilution technique. A hierarchical clustering method was used to construct dendrograms that were illustrated in heatmaps, which provided population-level information on clinically important bacterial species and their AMR patterns. Additionally, this study provides updated local AMR information for Illinois veterinarians to aid them in choosing effective drugs for therapy of canine UTIs. Our results are relevant to countries with similar antimicrobial treatment policies of UTIs, especially the North American region and other developed countries (33, 34).

### Prevalence of Major Urinary Bacterial Pathogens

The most frequent bacteria isolated from urine samples submitted from suspect UTI cases were *Escherichia coli, Staphylococcus pseudintermedius, Proteus mirabilis, Enterococcus faecalis, Streptococcus canis*, and *Enterococcus faecium*. These results correspond to previous studies from North America, Europe, and New Zealand that evaluated major bacteria isolated from canine urine samples and found *Staphylococcus* spp among the Gram-positive bacteria, and *E. coli* among Gram-negative bacteria as the most common (11, 13, 23, 35–38).

### Antimicrobial Resistance and Multidrug Resistance of Gram-Positive Bacterial Isolates

Assessing the heatmap of *Staphylococcus pseudintermedius* isolates, we showed that around 25% of isolates were susceptible to all antimicrobials tested. In addition, the second cluster was resistant to most of the antimicrobials tested except for rifampin, vancomycin, nitrofurantoin, and showed a low proportion of resistance to gentamicin and chloramphenicol. This cluster included all *Staphylococcus pseudintermedius* isolates that were resistant to oxacillin (27.08%) with MIC breakpoint ≥ 0.5 μg/ml defined as MRSP (39, 40). This result agrees with a previous European study, which described the prevalence of MRSP from canine urine samples ranging from 1.15 to 50% (37). It is known that MRSP isolates are frequently MDR, and the distribution of MDR isolates might differ geographically (41, 42). In our study, in addition to being resistant to β-lactam antibiotics, the MRSP isolates were resistant to fluoroquinolones, folate pathway antagonists, tetracyclines, lincosamides, and macrolides. As MRSP isolates were previously isolated from infected animals and their owner (19), our findings emphasize the importance of client education to prevent the zoonotic transmission of MRSP. Moreover, to prevent treatment failures of UTI in dogs, culture and susceptibility tests before initiation of treatment are warranted.

We identified a high prevalence of resistance to amoxicillin-clavulanic acid (27.08%) and trimethoprim-sulfamethoxazole (31.94%) among the *Staphylococcus pseudintermedius* isolates. This result is in agreement with previous studies that described a proportion of resistance to amoxicillin-clavulanic acid between 0.5 and 30.43% (3, 13, 43–45), and a proportion of resistance to trimethoprim-sulfamethoxazole between 2.77 and 63% among *Staphylococcus pseudintermedius* isolates (3, 13, 37, 43–45). In non-MRSP isolates, a high proportion of isolates were susceptible to cephalosporins, which could be used as empirical treatment. Our findings have clinical relevance as amoxicillin-clavulanic acid and trimethoprim-sulfamethoxazole are considered as the first-choice antimicrobials for empirical UTI treatments of dogs (20), and cephalosporins are generally effective treatment options for *Staphylococcus pseudintermedius* infections (46). These findings highlight the importance of conducting an antimicrobial susceptibility test before starting UTI treatments. In this study, we did not have access to antimicrobial treatment data; however, the over or misuse of these antimicrobials might impact the development of AMR to these drugs.

Our results showed that all *Staphylococcus pseudintermedius* isolates, including MRSP, were susceptible to rifampin, vancomycin, and nitrofurantoin. According to the International Society for Companion Animal Infectious Diseases (ISCAID) guidelines, nitrofurantoin could be the next possible option to treat UTI in dogs caused by MRSP. Meanwhile, both rifampin and vancomycin were not included in the ISCAID guidelines for UTI treatment in dogs (20), as rifampin is known for its potentially hepatotoxic effect in dogs (41) and excreted in an inactive form in the urine (47). Meanwhile, vancomycin can cause kidney injury (48, 49). More importantly, vancomycin is considered as the last resort option to treat Gram-positive bacterial infections in humans and animals (50, 51). There are public health concerns associated with the emergence of vancomycin-resistant enterococci (VRE) (50) and vancomycin-resistant *Staphylococcus aureus* (52). It is encouraging that our study did not identify any vancomycin-resistant isolates among Gram-positive bacteria. In addition, veterinarians should inform pet owners about the zoonotic transmission potential of this pathogen, as several reports indicated the transmission of *Staphylococcus pseudintermedius* from family dogs to humans with underlying medical conditions (53–55).

In our study, *Streptococcus canis* isolates showed a high prevalence of resistance to commonly used drugs to treat UTI in dogs, such as enrofloxacin, and marbofloxacin. This result was in contrast with the findings of a previous European study that found a high proportion of isolates susceptible to fluoroquinolones (3). Concurrently, our study showed similar results to previous studies from Europe and Canada that reported the majority of *Streptococcus canis* isolates were susceptible to penicillin, ampicillin, and amoxicillin-clavulanic acid (3, 56, 57). Differences in AMR patterns might be related to the geographical variations in antimicrobial use practices. Therefore, clinicians in Illinois and the US generally may consider penicillins as the first-line choice when treating UTI in dogs caused by *Streptococcus canis*.

When we compared the AMR patterns between *Staphylococcus pseudintermedius* and *Streptococcus canis* isolates, we found that the *Streptococcus canis* isolates had a higher odd of being resistant to enrofloxacin and marbofloxacin. Previous studies reported among *Staphylococcus pseudintermedius* isolates a prevalence of resistance to fluoroquinolones between 1.54% in Sweden and 42.11% in Italy (37). While among the *Streptococcus canis* isolates, the prevalence of resistance to fluoroquinolones was 96% in Australia (44) and 2% in New Zealand (13). Our findings are important for clinicians as fluoroquinolones are reserved to be used for recurrent UTIs in dogs with MDR bacteria as they are excreted through urine (20). Hence, the drugs should not be recommended as a first-line option for UTI treatment caused by these Gram-positive bacteria.

When comparing the *Enterococcus faecalis* and *Enterococcus faecium* isolates, different AMR patterns were observed. Our findings showed that *Enterococcus faecalis* isolates were susceptible to ampicillin and penicillin, while *Enterococcus faecium* isolates had an extremely high prevalence of resistance to these antimicrobials. These results suggest that ampicillin and penicillin could be considered as a first-line option to treat UTI caused by *Enterococcus faecalis*; however, it should not be recommended for UTI caused by *Enterococcus faecium*. On that account, these findings emphasize the importance of performing bacterial culture and antimicrobial susceptibility tests before initiating treatment.

### Antimicrobial Resistance and Multidrug Resistance of Gram-Negative Bacterial Isolates

Among Gram-negative bacteria, *E. coli* was the most prevalent in our study, comprising 45.58% of the total bacterial isolates. This finding agrees with previous studies conducted in the United Kingdom and North America (11, 23). Our study showed that overall, *E. coli* had a moderate to low prevalence of resistance to most of the antimicrobials except for ampicillin (31.42%). These results were consistent with previous studies, which reported a high prevalence of resistance to ampicillin among *E. coli* isolates (3, 58). When evaluating the AMR clustering dendrogram of *E. coli* isolates, we identified a large cluster that included approximately 56% of isolates that were susceptible to all antimicrobials tested; in addition to that, our study observed only 0.27% isolates that were resistant to imipenem, which was encouraging. A second cluster included isolates that were resistant to ampicillin, amoxicillin-clavulanic acid, and cephalosporins (i.e., cephalexin, cefpodoxime, cefovecin, cefazolin, ceftazidime). Our results showed that multidrug resistance was detected in 23.22% of *E. coli* isolates, which was lower than the previously reported prevalence of 43.3% in Japan (59), 28.9% in the United States (24), and 66.8% in Poland (60).

When we compared the AMR patterns between *E. coli* and Proteus mirabilis isolates, *E. coli* isolates had a higher odd of resistance to ampicillin. This result is in agreement with a previous study in Europe that showed a high prevalence of resistance to ampicillin and detected a higher level of antimicrobial resistance in E. coli isolates compared to other bacteria of the *Enterobacteriaceae* family (3). In the ISCAID guideline, amoxicillin is recommended as the first-line option for bacterial UTI treatment in dogs, and its antimicrobial susceptibility can be predicted by evaluating the susceptibility of isolates to ampicillin (20). Overall, our results of the E. coli and Proteus mirabilis AMR patterns support the finding of previous studies that showed low resistance of the isolates to amoxicillin-clavulanic acid, amikacin, and gentamicin (3, 56). Considering the nephrotoxicity characteristic of amikacin and gentamicin, these drugs should be reserved for complicated UTIs with careful application. Thus, amoxicillin-clavulanic acid might be an appropriate first option for empirical treatment of UTI in dogs for *E. coli* and Proteus mirabilis in Illinois, US. In case of resistance to this drug, trimethoprim-sulfamethoxazole and fluoroquinolones should be considered as the next choices to treat UTI in dogs.

Before interpreting our study results, a few limitations should be noted. We might overestimate the prevalence of AMR to individual and multiple antimicrobials as canine urine samples were submitted from UTI cases that might have already been treated with antimicrobials. Recurrent cases might also be overrepresented in our study as we evaluated urine samples that were submitted to a veterinary referral laboratory. Future studies should evaluate the impact of previous antimicrobial use and clinical status of dogs on the emergence of AMR and MDR in urinary bacterial pathogens. Additionally, in our study, urine samples were collected through cystocentesis, catheterization, or free catch that could affect the determination of significant bacteria that cause the real UTI in our study results. Consideration of the collection method is important in evaluating the need for antimicrobial therapy as low levels of bacteria from free catch samples may represent contamination and is not indicative of a need for antimicrobial therapy. Cystocentesis is the most reliable and recommended method for urine sample collection for bacterial culture to prevent contamination of samples (10). However, cystocentesis may not always be feasible in some clinical settings because it requires client consent and depends on the patient's condition. As we focused on analyzing the overall AMR patterns of main urinary bacterial species, we included all culture-positive isolates regardless of their method of collection. Some of our AMR patterns of Gram-positive and Gram-negative bacteria should be interpreted with caution as not all MIC breakpoints were available for bacteria causing UTIs in dogs. If MIC breakpoints were not available, we used breakpoints defined for other infection sites (i.e., skin, wound) in dogs, or we applied MIC breakpoints defined for human bacterial infections. Lastly, UTI clinical outcomes after antimicrobial treatment might not correlate completely with the susceptibility results of pathogens, as certain antimicrobials can achieve high concentrations in the urine, and they could be effective.

## Conclusion

We provided recent local information on the prevalence of major Gram-positive and Gram-negative bacteria that were isolated from canine urine samples from suspected UTI cases that were submitted to a veterinary diagnostic laboratory in Illinois, US. *Escherichia coli, Staphylococcus pseudintermedius, Proteus mirabilis, Enterococcus faecalis, Streptococcus canis*, and *Enterococcus faecium* were the most prevalent bacteria isolated from canine urine samples. The prevalence of AMR among major Gram-positive bacteria toward first-line antimicrobial choices to treat UTI in dogs such as amoxicillin-clavulanic acid and trimethoprim-sulfamethoxazole was high in *Staphylococcus pseudintermedius* isolates, which suggest that antimicrobial use practices might have an impact on the development of resistance to these antimicrobials. A high proportion of *Staphylococcus pseudintermedius* and *Klebsiella pneumoniae* isolates were resistant to fluoroquinolones and 3rd generation cephalosporins. Within the Gram-negative bacteria, *E. coli* isolates presented a moderate to low prevalence of resistance toward all antimicrobials tested. Since dogs could become the reservoirs of MDR bacteria that may be transmitted to humans, veterinarians should inform dog owners about the potential zoonotic transmission risk of these pathogens.

The findings of this study can assist clinicians in their antimicrobial choices when treating UTI and highlight the importance of collecting urine samples and conducting bacterial culture and antimicrobial susceptibility tests before starting UTI treatments to prevent the development of MDR bacteria. Continuous monitoring of the AMR patterns of clinically important bacterial urinary pathogens is warranted to identify emerging MDR strains.

## Data Availability Statement

The original contributions presented in the study are included in the article/Supplementary Material, further inquiries can be directed to the corresponding authors.

## Ethics Statement

Ethical review and approval was not required for the animal study because de-identified laboratory data were analyzed to keep clients' and their animals' information confidential. The bacterial culture and antimicrobial sensitivity data cannot be linked to patient or owner information. Written informed consent for participation was not obtained from the owners because de-identified laboratory data were analyzed to keep clients' and their animals' information confidential. The bacterial culture and antimicrobial sensitivity data cannot be linked to patient or owner information.

## Author Contributions

SY and CV: study design, data analysis, and writing—original draft. CV and C-CH: resources. SY, CV, C-CH, and CM: writing—review and editing. SY: visualization. CV: supervision. CV and C-CH: project administration. All authors contributed to the article and approved the submitted version.

## Funding

Companion Animal Research Grant Program, College of Veterinary Medicine, University of Illinois at Urbana-Champaign.

## Conflict of Interest

The authors declare that the research was conducted in the absence of any commercial or financial relationships that could be construed as a potential conflict of interest.

## Publisher's Note

All claims expressed in this article are solely those of the authors and do not necessarily represent those of their affiliated organizations, or those of the publisher, the editors and the reviewers. Any product that may be evaluated in this article, or claim that may be made by its manufacturer, is not guaranteed or endorsed by the publisher.

## Abbreviations

AMR: antimicrobial resistance
CLSI: Clinical and Laboratory Standards Institute
MALDI-TOF MS: matrix-assisted laser desorption ionization-time of flight mass spectrometry
MIC: minimum inhibition concentration
MDR: multidrug-resistant
US: United States of America
UTI: urinary tract infections.

## References

[1] Amphaiphan C Yano T Som-in M Kungwong P Wongsawan K Pusoonthornthum R. Antimicrobial drug resistance profile of isolated bacteria in dogs and cats with urologic problems at Chiang Mai University Veterinary Teaching Hospital, Thailand (2012–2016). Zoonoses Public Health. (2021) 68:452–63. 10.1111/zph.12832 33844465

[2] Darwich L Seminati C Burballa A Nieto A Durán I Tarradas N. Antimicrobial susceptibility of bacterial isolates from urinary tract infections in companion animals in Spain. Vet Rec. (2021) 188:e60. 10.1002/vetr.60 33960452

[3] Li Y Fernández R Durán I Molina-López RA Darwich L. Antimicrobial resistance in bacteria isolated from cats and dogs from the Iberian Peninsula. Front Microbiol. (2021) 11621597. 10.3389/fmicb.2020.621597 33584590 PMC7874003

[4] Johnstone T. A clinical approach to multidrug-resistant urinary tract infection and subclinical bacteriuria in dogs and cats. N Z Vet J. (2020) 68:69–83. 10.1080/00480169.2019.1689196 31707934

[5] Scott Weese J. Antimicrobial resistance in companion animals. Animal Health Research Reviews/Conference of Research Workers in Animal Diseases. (2008) 9:169–176. 10.1017/S1466252308001485 18983722

[6] Pomba C Rantala M Greko C Baptiste KE Catry B van Duijkeren E. Public health risk of antimicrobial resistance transfer from companion animals. J Antimicrob Chemother. (2017) 72:957–68. 10.1093/jac/dkw481 27999066

[7] Boothe D Smaha T Carpenter DM Shaheen B Hatchcock T. Antimicrobial resistance and pharmacodynamics of canine and feline pathogenic *E. coli* in the united states. J Am Anim Hosp Assoc. (2012) 48:379–89. 10.5326/JAAHA-MS-5805 23033458

[8] Conner JG Smith J Erol E Locke S Phillips E Carter CN. Temporal trends and predictors of antimicrobial resistance among *Staphylococcus spp*. isolated from canine specimens submitted to a diagnostic laboratory. PLoS ONE. (2018) 13:e0200719. 10.1371/journal.pone.0200719 30067775 PMC6070192

[9] Cummings KJ Aprea VA Altier C. Antimicrobial resistance trends among canine *Escherichia coli* isolates obtained from clinical samples in the northeastern USA. 2004-2011. Can Vet J. (2015) 56:393–8. 10.1089/fpd.2013.1605 25829560 PMC4357913

[10] Bartges JW. Diagnosis of urinary tract infections. Vet Clin N Am Small Anim Pract. (2004) 34:923–33. 10.1016/j.cvsm.2004.03.001 15223209

[11] Hall JL Holmes MA Baines SJ. Prevalence and antimicrobial resistance of canine urinary tract pathogens. Vet Rec. (2013) 173:549. 10.1136/vr.101482 24158327

[12] Hernando E Vila A D'Ippolito P Rico AJ Rodon J Roura X. Prevalence and characterization of urinary tract infection in owned dogs and cats from Spain. Top Companion Anim Med. (2021) 43:100512. 10.1016/j.tcam.2021.100512 33484889

[13] McMeekin CH Hill KE Gibson IR Bridges JP Benschop J. Antimicrobial resistance patterns of bacteria isolated from canine urinary samples submitted to a New Zealand veterinary diagnostic laboratory between 2005–2012. N Z Vet J. (2017) 65:99–104. 10.1080/00480169.2016.1259594 27842208

[14] Tyson GH Li C Ceric O Reimschuessel R Cole S Peak L. Complete genome sequence of a carbapenem-resistant *Escherichia coli* isolate with bla NDM-5 from a dog in the United States. Microbiol Resour Announc. (2019) 8:e00872–19. 10.1128/MRA.00872-19 31439705 PMC6706697

[15] Rubin J Walker RD Blickenstaff K Bodeis-Jones S Zhao S. Antimicrobial resistance and genetic characterization of fluoroquinolone resistance of Pseudomonas aeruginosa isolated from canine infections. Vet Microbiol. (2008) 131:164–72. 10.1016/j.vetmic.2008.02.018 18395369

[16] Harada K Arima S Niina A Kataoka Y Takahashi T. Characterization of Pseudomonas aeruginosa isolates from dogs and cats in Japan: current status of antimicrobial resistance and prevailing resistance mechanisms. Microbiol Immunol. (2012) 56:123–7. 10.1111/j.1348-0421.2011.00416.x 22188523

[17] Grönthal T Eklund M Thomson K Piiparinen H Sironen T Rantala M. Antimicrobial resistance in *Staphylococcus pseudintermedius* and the molecular epidemiology of methicillin-resistant *S. pseudintermedius* in small animals in Finland. J Antimicrob Chemother. (2017) 72:1021–30. 10.1093/jac/dkx086 28065889 PMC5400095

[18] Smith JT Amador S McGonagle CJ Needle D Gibson R Andam CP. Population genomics of *Staphylococcus pseudintermedius* in companion animals in the United States. Commun Biol. (2020) 3:282. 10.1038/s42003-020-1009-y 32503984 PMC7275049

[19] Soedarmanto I Kanbar T Ülbegi-Mohyla H Hijazin M Alber J Lämmler C. Genetic relatedness of methicillin-resistant *Staphylococcus pseudintermedius* (MRSP) isolated from a dog and the dog owner. Res Vet Sci. (2011) 91:e25–7. 10.1016/j.rvsc.2011.01.027 21353270

[20] Weese JS Blondeau J Boothe D Guardabassi LG Gumley N Papich M. International Society for Companion Animal Infectious Diseases (ISCAID) guidelines for the diagnosis and management of bacterial urinary tract infections in dogs and cats. Vet J. (2019) 247:8–25. 10.1016/j.tvjl.2019.02.008 30971357

[21] Guardabassi L Schwarz S Lloyd DH. Pet animals as reservoirs of antimicrobial-resistant bacteria. J Antimicrob Chemother. (2004) 54:321–32. 10.1093/jac/dkh332 15254022

[22] Thompson MF Litster AL Platell JL Trott DJ. Canine bacterial urinary tract infections: new developments in old pathogens. Vet J. (2011) 190:22–7. 10.1016/j.tvjl.2010.11.013 21239193

[23] Wong C Epstein SE Westropp JL. Antimicrobial susceptibility patterns in urinary tract infections in dogs (2010–2013). J Vet Intern Med. (2015) 29:1045–52. 10.1111/jvim.13571 26133165 PMC4895361

[24] Shaheen BW Boothe DM Oyarzabal OA Smaha T. Antimicrobial resistance profiles and clonal relatedness of Canine and Feline *Escherichia coli* pathogens expressing multidrug resistance in the United States. J Vet Intern Med. (2010) 24:323–30. 10.1111/j.1939-1676.2009.0468.x 20102505

[25] Jackson CR Fedorka-Cray PJ Davis JA Barrett JB Frye JG. Prevalence, species distribution and antimicrobial resistance of enterococci isolated from dogs and cats in the United States. J Appl Microbiol. (2009) 107:1269–78. 10.1111/j.1365-2672.2009.04310.x 19486402

[26] Thungrat K Price SB Carpenter DM Boothe DM. Antimicrobial susceptibility patterns of clinical *Escherichia coli* isolates from dogs and cats in the United States: January 2008 through January 2013. Vet Microbiol. (2015) 179:287–95. 10.1016/j.vetmic.2015.06.012 26165272

[27] Verma A Carney K Taylor M Amsler K Morgan J Gruszynski K. Occurrence of potentially zoonotic and cephalosporin resistant enteric bacteria among shelter dogs in the Central and South-Central Appalachia. BMC Vet Res. (2021) 17:313. 10.1186/s12917-021-03025-2 34563197 PMC8467218

[28] CLSI. (2020). Performance Standards for Antimicrobial Disk and Dilution Susceptibility Tests for Bacteria Isolated From Animals. 5th Edn. (5 th). Clinical and Laboratory Standards Institute. Availabl online at: http://clsivet.org/GetDoc.aspx?doc=CLSI VET01S ED5:2020andscope=user.

[29] CLSI. CLSI. Performance Standards for Antimicrobial Disk and Dilution Susceptibility Tests for Bacteria Isolated From Animals. 5th Edn. (2021). CLSI supplement VET01Clinical S, and Laboratory Standards Institute. Availabl online at: http://em100.edaptivedocs.net/GetDoc.aspx?doc=CLSI M100 ED31:2021andscope=user.

[30] Authority EFS. The European Union Summary Report on Antimicrobial Resistance in zoonotic and indicator bacteria from humans, animals and food in 2018/2019. EFSA J. (2021) 19:e06490. 10.2903/j.efsa.2021.6490 33868492 PMC8040295

[31] Magiorakos AP Srinivasan A Carey RB Carmeli Y Falagas ME Giske CG. Multidrug-resistant, extensively drug-resistant and pandrug-resistant bacteria: An international expert proposal for interim standard definitions for acquired resistance. Clin Microbiol Infect. (2012) 18:268–81. 10.1111/j.1469-0691.2011.03570.x 21793988

[32] Murtagh F Legendre P. Ward's hierarchical agglomerative clustering method: which algorithms implement ward's criterion? J Classif. (2014) 31:274–95. 10.1007/s00357-014-9161-z

[33] Jessen LR Sørensen TM Bjornvad CR Nielsen SS Guardabassi L. Effect of antibiotic treatment in canine and feline urinary tract infections: a systematic review. Vet J. (2015) 203:270–7. 10.1016/j.tvjl.2014.12.004 25634080

[34] Marques C Belas A Franco A Aboim C Gama LT Pomba C. Increase in antimicrobial resistance and emergence of major international high-risk clonal lineages in dogs and cats with urinary tract infection: 16 year retrospective study. J Antimicrob Chemother. (2018) 73:377–84. 10.1093/jac/dkx401 29136156 PMC5890753

[35] Ball KR Rubin JE Chirino-Trejo M Dowling PM. Antimicrobial resistance and prevalence of canine uropathogens at the Western College of Veterinary Medicine Veterinary Teaching Hospital, 2002-2007. Can Vet J. (2008) 49:985–90. http://www.ncbi.nlm.nih.gov/pubmed/19119366. 19119366 PMC2553511

[36] Blondeau JM Fitch SD. In vitro killing of canine urinary tract infection pathogens by ampicillin, cephalexin, marbofloxacin, pradofloxacin, and trimethoprim/sulfamethoxazole. Microorganisms. (2021) 9:2279. 10.3390/microorganisms9112279 34835405 PMC8619264

[37] Marques C Gama LT Belas A Bergström K Beurlet S Briend-Marchal A. European multicenter study on antimicrobial resistance in bacteria isolated from companion animal urinary tract infections. BMC Vet Res. (2016) 12:213. 10.1186/s12917-016-0840-3 27658466 PMC5034465

[38] Teh H. A review of the current concepts in canine urinary tract infections. Aust Vet J. (2022) 100:56–62. 10.1111/avj.13127 34775603

[39] Bemis DA Jones RD Frank LA Kania SA. Evaluation of susceptibility test breakpoints used to predict mecA-mediated resistance in *Staphylococcus pseudintermedius* isolated from dogs. J Vet Diagn Invest. (2009) 21:53–8. 10.1177/104063870902100108 19139501

[40] Wu MT Burnham CAD Westblade LF Bard JD Lawhon SD Wallace MA. Evaluation of oxacillin and cefoxitin disk and MIC breakpoints for prediction of methicillin resistance in human and veterinary isolates of *Staphylococcus intermedius* group. J Clin Microbiol. (2016) 54:535–42. 10.1128/JCM.02864-15 26607988 PMC4767974

[41] Frank LA Loeffler A. Meticillin-resistant *Staphylococcus pseudintermedius*: clinical challenge and treatment options. Vet Dermatol. (2012) 23:283–91, e56. 10.1111/j.1365-3164.2012.01047.x 22486942

[42] Rubin JE Gaunt MC. Urinary tract infection caused by methicillin-resistant *Staphylococcus pseudintermedius* in a dog. Can Vet J. (2011) 52:169–72. 21532822 PMC3022454

[43] Penna B Varges R Martins R Martins G Lilenbaum W. In vitro antimicrobial resistance of staphylococci isolated from canine urinary tract infection. Can Vet J. (2010) 51:738–42. 20885826 PMC2885114

[44] Scarborough R Bailey K Galgut B Williamson A Hardefeldt L Gilkerson J. Use of local antibiogram data and antimicrobial importance ratings to select optimal empirical therapies for urinary tract infections in dogs and cats. Antibiotics. (2020) 9:924. 10.3390/antibiotics9120924 33353226 PMC7766990

[45] Windahl U Holst BS Nyman A Grönlund U Bengtsson B. Characterisation of bacterial growth and antimicrobial susceptibility patterns in canine urinary tract infections. BMC Vet Res. (2014) 10:217. 10.1186/s12917-014-0217-4 25249356 PMC4180317

[46] Westermeyer RR Roy AF Mitchell MS Merchant SR. *In vitro* comparison of *Staphylococcus pseudintermedius* susceptibility to common cephalosporins used in dogs. Vet Ther. (2010) 11:E1–9. 20960413

[47] Plumb DC. Plumb's Veterinary Drug Handbook. (2018). Desk. John Wiley and Sons.

[48] DeStefano IM Wayne AS Rozanski EA Babyak JM. Parenterally administered vancomycin in 29 dogs and 7 cats (2003–2017). J Vet Intern Med. (2019) 33:200–7. 10.1111/jvim.15357 30499215 PMC6335575

[49] Pais GM Liu J Zepcan S Avedissian SN Rhodes NJ Downes KJ. Vancomycin-induced kidney injury: animal models of toxicodynamics, mechanisms of injury, human translation, and potential strategies for prevention. Pharmacotherapy. (2020) 40:438–54. 10.1002/phar.2388 32239518 PMC7331087

[50] Ahmed MO Baptiste KE. Vancomycin-resistant enterococci: a review of antimicrobial resistance mechanisms and perspectives of human and animal health. Microbial Drug Resist. (2018) 24:590–606. 10.1089/mdr.2017.0147 29058560

[51] Mühlberg E Umstätter F Kleist C Domhan C Mier W Uhl P. Renaissance of vancomycin: approaches for breaking antibiotic resistance in multidrug-resistant bacteria. Can J Microbiol. (2020) 66:11–6. 10.1139/cjm-2019-0309 31545906

[52] Mahros MA Abd-Elghany SM Sallam KI. Multidrug-, methicillin-, and vancomycin-resistant *Staphylococcus aureus* isolated from ready-to-eat meat sandwiches: an ongoing food and public health concern. Int J Food Microbiol. (2021) 346:109165. 10.1016/j.ijfoodmicro.2021.109165 33770679

[53] Blondeau LD Rubin JE Deneer H Kanthan R Morrison B Sanche S. Persistent infection with *Staphylococcus pseudintermedius* in an adult oncology patient with transmission from a family dog. J Chemother. (2020) 32:151–5. 10.1080/1120009X.2020.1735142 32124685

[54] Blondeau LD Deutscher M Rubin JE Deneer H Kanthan R Sanche S. Urinary tract infection in a human male patient with *Staphylococcus pseudintermedius* transmission from the family dog. J Chemother. (2021) 34:133–6. 10.1080/1120009X.2021.1995251 34747350

[55] Carroll KC Burnham CAD Westblade LF. From canines to humans: clinical importance of *Staphylococcus pseudintermedius*. PLoS Pathog. (2021) 17:e1009961. 10.1371/journal.ppat.1009961 34855921 PMC8638991

[56] Awosile BB Mcclure JT Saab ME Heider LC. Antimicrobial resistance in bacteria isolated from cats and dogs from the Atlantic Provinces, Canada from 1994–2013. Can Vet J. (2018) 59:885–93. 30104781 PMC6049328

[57] Pedersen K Pedersen K Jensen H Finster K Jensen VF Heuer OE. Occurrence of antimicrobial resistance in bacteria from diagnostic samples from dogs. J Antimicrob Chemother. (2007) 60:775–81. 10.1093/jac/dkm269 17644533

[58] LeCuyer TE Byrne BA Daniels JB Diaz-Campos DV Hammac GK Miller CB. Population structure and antimicrobial resistance of canine uropathogenic *Escherichia coli*. J Clin Microbiol. (2018) 56:e00788–18. 10.1128/JCM.00788-18 29997200 PMC6113483

[59] Harada K Niina A Nakai Y Kataoka Y Takahashi T. Prevalence of antimicrobial resistance in relation to virulence genes and phylogenetic origins among urogenital *Escherichia coli*. isolates from dogs and cats in Japan. Am J Vet Res. (2012) 73:409–17. 10.2460/ajvr.73.3.409 22369535

[60] Rzewuska M Czopowicz M Kizerwetter-wida M Chrobak D Błaszczak B Binek M. Multidrug resistance in *Escherichia coli* strains isolated from infections in dogs and cats in Poland (2007–2013). Sci World J. (2015) 2015:408205. 10.1155/2015/408205 25667937 PMC4312638

