# Supplementary material for: Antimicrobial Resistance in Bacteria Isolated From Canine Urine Samples Submitted to a Veterinary Diagnostic Laboratory, Illinois, United States
Source: Front Vet Sci. 2022 May 4;9:867784. doi: 10.3389/fvets.2022.867784 (PMC9114880; doi:10.3389/fvets.2022.867784)
Supplement: Supplementary file 1 [file Table_1.DOCX]

**Supplementary Table 1. Comparison of antimicrobial resistance patterns between *Staphylococcus pseudintermedius* and *Streptococcus canis* isolates**

| Antimicrobial agents^a^ | Coefficients (95 % CI)^b^ | Odds ratio | P-value^c^ |
| --- | --- | --- | --- |
| ENRO | -1.52 (-2.21, -0.83) | 0.22 | < 0.001 |
| MAR | -1.23 (-1.90, -0.56) | 0.29 | < 0.001 |
| ERY | 1.51 (0.53, 2.5) | 4.54 | 0.002 |
| CLI | 1.54 (0.56, 2.53) | 4.68 | 0.002 |

^a^ ENRO, enrofloxacin; MAR, marbofloxacin; ERY, Erythromycin; CLI, Clindamycin; ^b^ CI, confidence interval; ^c^ Statistically significant at P ≤ 0.05.

**Supplementary Table 2. Comparison of antimicrobial resistance patterns between *E. coli* and *Proteus mirabilis* isolates**

| Antimicrobial agents^a^ | Coefficients (95 % CI)^b^ | Odds ratio | P-value^c^ |
| --- | --- | --- | --- |
| GEN | -1.22 (-2.24, -0.20) | 0.3 | 0.02 |
| AUG2 | 1.71 (0.52, 2.89) | 5.51 | 0.005 |
| FAZ | 1.02 (0.07, 1.97) | 2.78 | 0.03 |
| FOV | 2.06 (0.63, 3.49) | 7.86 | 0.005 |
| POD | 2.00 (0.57, 3.43) | 7.37 | 0.006 |
| LEX | 2.04 (0.61, 3.47) | 7.69 | 0.005 |
| IMI | -2.84 (-5.05, -0.64) | 0.06 | 0.01 |
| AMP | 0.99 (0.36, 1.61) | 2.68 | 0.002 |
| CHL | -0.60 (-1.16, -0.04) | 0.55 | 0.04 |

^a^ GEN, gentamicin; AUG2, amoxicillin-clavulanic acid; FAZ, cefazolin; POD, cefpodoxime; LEX, cephalexin; IMI, imipenem; AMP, ampicillin; CHL, chloramphenicol; ^b^ CI, confidence interval; ^c^ Statistically significant at P ≤ 0.05.
